# Factors affecting irreversible inhibition of EGFR and influence of chirality on covalent binding

**DOI:** 10.1038/s42004-025-01501-6

**Published:** 2025-04-09

**Authors:** Pasquale A. Morese, Ayaz Ahmad, Mathew P. Martin, Richard A. Noble, Sara Pintar, Lan Z. Wang, Shangze Xu, Andrew Lister, Richard A. Ward, Agnieszka K. Bronowska, Martin E. M. Noble, Hannah L. Stewart, Michael J. Waring

**Affiliations:** 1 Cancer Research Horizons Newcastle Drug Discovery Unit, Chemistry, School of Natural and Environmental Sciences, Bedson Building, Newcastle University, Newcastle upon Tyne, UK; 2 Chemistry, School of Natural and Environmental Sciences, Bedson Building, Newcastle University, Newcastle upon Tyne, UK; 3 Cancer Research Horizons Newcastle Drug Discovery Unit, Translational and Clinical Research Institute, Paul O’Gorman Building, Newcastle University, Newcastle upon Tyne, UK; 4 Oncology iMed, R&D, AstraZeneca, Cambridge, UK

**Keywords:** Drug discovery and development, X-ray crystallography, Bioconjugate chemistry, Mechanism of action

## Abstract

The discovery of targeted covalent inhibitors is of increasing importance in drug discovery. Finding efficient covalent binders requires modulation of warhead reactivity and optimisation of warhead geometry and non-covalent interactions. Uncoupling the contributions that these factors make to potency is difficult and best practice for a testing cascade that is pragmatic and informative is yet to be fully established. We studied the structure-reactivity-activity relationships of a series of analogues of the EGFR inhibitor poziotinib with point changes in two substructural regions as well as variations in warhead reactivity and geometry. This showed that a simple probe displacement assay that is appropriately tuned in respect of timing and reagent concentrations can reveal structural effects on all three factors: non-covalent affinity, warhead reactivity and geometry. These effects include the detection of potency differences between an enantiomeric pair that differ greatly in their activity and their capacity to form a covalent bond. This difference is rationalised by X-ray crystallography and computational studies and the effect translates quantitatively into cellular mechanistic and phenotypic effects.

## Abstract (web summary)

Covalent inhibitors are a successful class of drugs, however, the optimization of targeted covalent inhibitors has challenges due to the need to increase non-covalent interactions and modulate reactivity. Here, the authors study the structure-reactivity-activity relationships of analogues of the EGFR inhibitor poziotinib, showing practical methods to characterize structure-activity relationships, providing insights into the origins of potency and highlighting the effect of chirality on covalent binding.

## Introduction

**Fig. 1:**
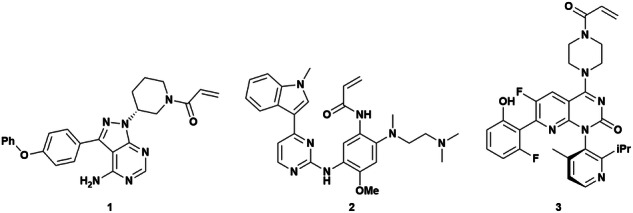
Structures of prominent TCIs: inhibitors of BTK (ibrutinib, **1**), EGFR (osimertinib, **2**) and mutant kRAS (sotorasib, **3**).

Targeted covalent inhibitors (TCIs) have undergone a renaissance in recent years, prompted in no small part by the clinical success of BTK^1^, EGFR^2^ and, more recently, kRAS G12C^3^ inhibitors in oncology (Fig. 1). TCIs offer numerous advantages for drug discovery since they can exhibit significantly increased binding energy, often by irreversible complex formation, and hence reduce the effect of competition for endogenous ligands^4,5^. The formation of covalent interactions with specific protein residues can give increased selectivity and provide a means of drugging less tractable binding sites. Additionally, targeting of specific nucleophilic residues, most commonly cysteine^6^, with electrophilic compounds is amenable to rational, structure-based drug design^7^.

Optimisation of TCIs does, however, present its challenges. It is critical to modulate the reactivity of the covalent warhead such that it can react with the target protein but is not so high that it reacts indiscriminately with other endogenous nucleophiles. A useful guide to optimal reactivity is to measure the glutathione (GSH) half-life of compounds, with an ideal reactivity window being between 30 and 120 minutes, as compared to marketed covalent inhibitors (**1**-**3**)^8^. Acrylamide moieties typically fall within this range and for this reason are perhaps the most commonly employed covalent warhead for cysteine-based TCIs.

Another key consideration in optimisation is to impart sufficient non-covalent affinity into lead compounds and not to rely overly on their reactivity to achieve potency. This involves forming additional non-covalent interactions and, more subtly, to ensure the binding pose of the encounter complex is optimal for the ensuing covalent bond-forming reaction^9^.

These factors can be hard to elucidate in a pragmatic manner. Ideally, compounds should be characterised by their *K*_I_ and *k*_inact_ data, but accurate determination of these parameters is intensive in time and resources and difficult to implement as a primary assay. Consequently, optimisation is often driven using assays that are configured for non-covalent inhibitors. IC_50_ values for TCIs derived from these assays are time dependent and in the purest sense, are considered meaningless^10^. However, their values can be useful pragmatically to derive structure-activity relationships (SAR) with important provisos: they should be determined at a consistent and appropriately chosen time point, interpreted alongside an assessment of reactivity (such as the GSH t_½_), and should compare compounds of similar reactivity. Often, an assessment of covalent binding, such as protein mass spectrometry, is incorporated into a test cascade to confirm covalent binding and, in some cases, the extent of labelling is also used to derive SAR.

**Fig. 2:**
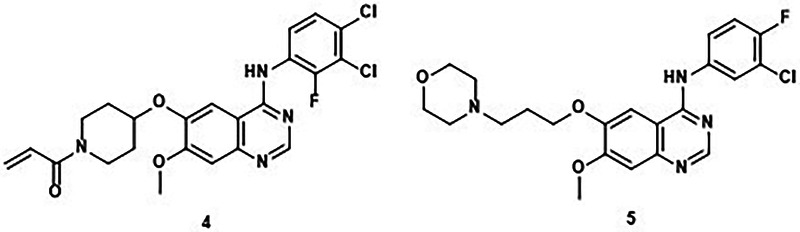
Structures of poziotinib **4** and gefitinib **5**, covalent and non covalent anilinoquinazoline-based EGFR inhibitors respectively.

Best practice for the approach to the design-make-test process for TCIs is not fully established. We studied a congeneric series of analogues of the anilinoquinazoline-based EGFR TCI poziotinib^11^ (**4**, Fig. 2) to explore some of the important factors relating to SAR generation and profiling of TCIs.

## Results and discussion

### TR-FRET probe

**Fig. 3:**
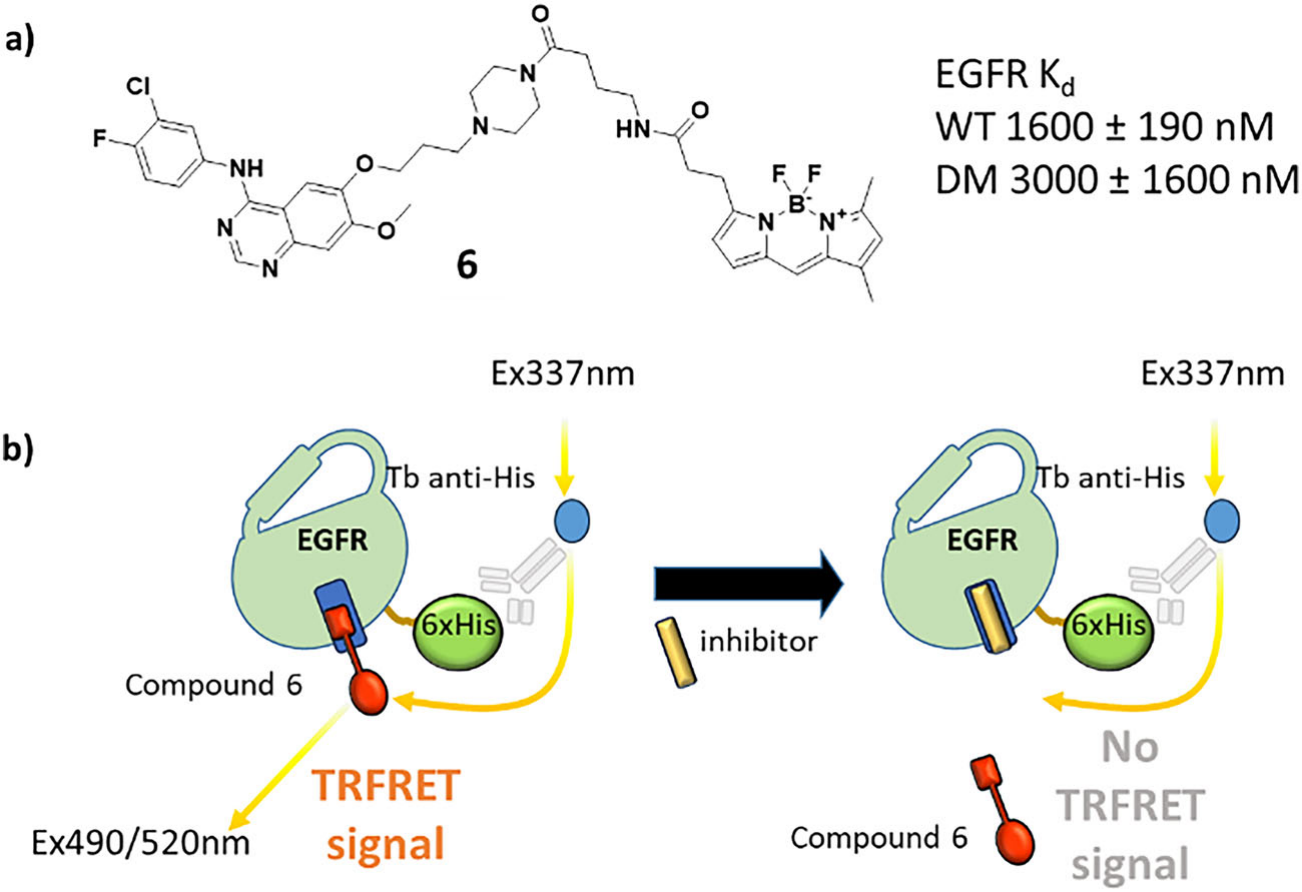
Development of the TR FRET based probe displacement assay. **a** Gefitinib derived fluorescent probe **6**; (**b**) Schematic overview of the assay.

To develop a suitable assay for EGFR, a fluorescent displacement probe for the active (ATP-binding) site based on the non-covalent anilinoquinazoline inhibitor gefitinib **5**^12^ was designed (Fig. 3). The binding mode of gefitinib, typical of an anilinoquinazoline-based kinase inhibitor, is well characterised, with the morpholine side-chain extending into the solvent exposed region where a variety of substituents are tolerated. Hence, this was an attractive position to incorporate a fluorophore. Accordingly, probe compound **6** was prepared, in which the morpholine was replaced by a piperazine and a BODIPY fluorophore was attached via a 4-aminobutanoate spacer (Fig. 3a). A related sapitinib-derived tracer has subsequently been described^13^. Compound **6** was employed in a time-resolved Förster-resonance energy transfer (TR-FRET) assay with EGFR bound to a terbium-containing antibody (Fig. 3b). In line with our previous work exploring the activity of anilinoquinazoline-based acrylamide TCIs^14^, the incubation time for the assay was set at 30 mins.

### Compound design and synthesis

**Fig. 4:**
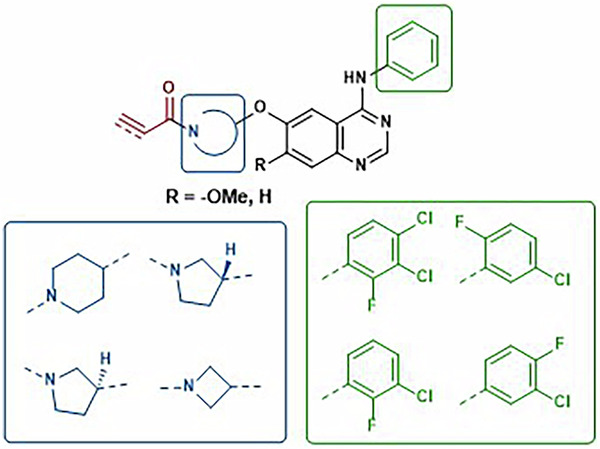
Deconstruction of the poziotinib structure to design analogues varying the warhead (red), cyclic amine spacer (blue), quinazoline substituent (R) and aniline (green).

A series of analogues of poziotinib were designed that had variations in the aniline and quinazoline substituents to explore differences in non-covalent affinity and to generate matched pairs for SAR analysis (Fig. 4). Variations in the cyclic amine spacer bearing the covalent warhead were introduced to explore the impact of changes in warhead geometry relative to the quinazoline scaffold. Acrylamide warheads were attached to these analogues as well as the more reactive propiolamides.

**Scheme 1:**
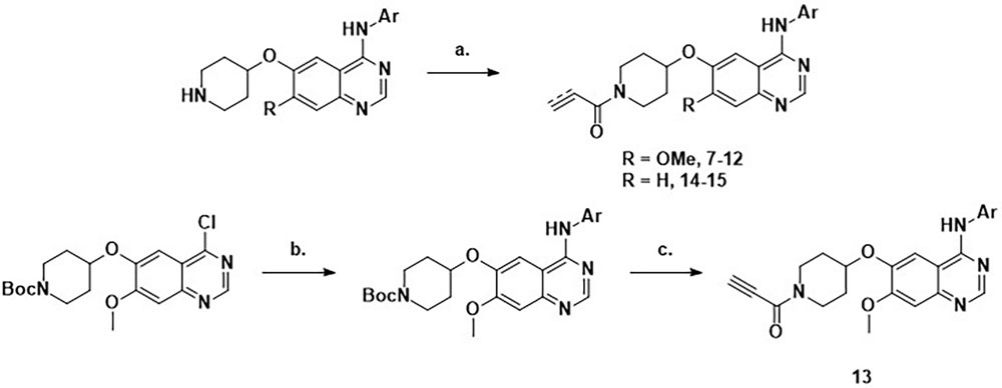
Synthesis of compounds. **7**-**15**. Reagents and conditions: a) Acryloyl chloride, NaHCO_3_, THF, 0 °C – r.t., 12 h (**7-9, 14:** 9–71%) or propiolic acid, HATU, DIPEA, DMA, rt, 2 h (**10-12, 15:** 37–75%); (**b**) NH_2_Ar, DMF, 75 °C, 1.5 h, 44%; (**c**) i. TFA, DCM, rt, 1 h, ii. propiolic acid, HATU, DIPEA, DMA, rt, 2 h, (**13**: 91%).

For exploration of the cyclic amine linker, commercially available 4-((3-chloro-4-fluorophenyl)amino)-7-methoxyquinazolin-6-ol was alkylated with the N-boc-protected mesylate of the appropriate cyclic amine. Subsequent deprotection and acylation gave rise to acrylamides 16-18 and propiolamides 19-21 (Scheme 1).

**Scheme 2:**
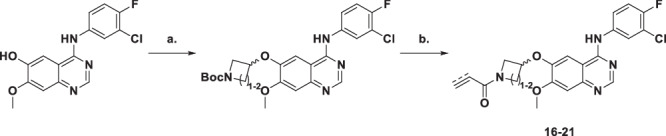
Synthesis of compounds 16-21. Reagents and conditions: (**a**) N-Boc-mesylate, K_2_CO_3_, DMF, 80 °C, 12 h (24–65%) or propiolic acid, HATU, DIPEA, DMA, rt, 2 h (**10-12, 15:** 37–75%); (**b**). i. TFA, DCM, rt, 1 h, ii. acryloyl chloride, NaHCO_3_, THF, 0 °C – r.t., 12 h (**16-18**: 13-42%) or propiolic acid, HATU, DIPEA, DMA, rt, 2 h, (**19-21**: 60–76%).

To separate the covalent and non-covalent contributions to binding, non-covalent analogues of **16** and **17** were synthesised by the alkylation of 4-((3-chloro-4-fluorophenyl)amino)-7-methoxyquinazolin-6-ol with the N-boc-protected pyrollidine mesylate. Subsequent deprotection and acylation gave rise to **22** and **23** (Scheme 2).

**Scheme 3:**
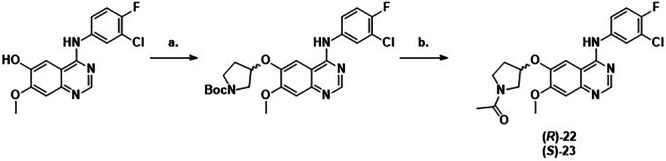
Synthesis of compounds 22 and 23. Reagents and conditions: (**a**) N-Boc-mesylate, K_2_CO_3_, DMF, 80 °C, 12 h (24-65%) or propiolic acid, HATU, DIPEA, DMA, rt, 2 h (**10-12, 15:** 37-75%); (**b**). i. TFA, DCM, rt, 1 h, ii. acetyl chloride, NaHCO_3_, THF, 0 °C – r.t., 12 h (**22-23**: 17-21%).

For variations in the aryl substituent, literature procedures^15^ gave rise to the appropriate piperidines, which were acylated to form acrylamides 7-9 and 14 or propiolamides 10-12 and 15. For the 4,5-dichloro-2-fluorophenyl analogue 13, the Boc-protected piperidine quinazoline was substituted then deprotected and acylated to give propiolamide 13 (Scheme 3).

### Structure-activity relationships

The compounds were tested in the TR FRET assay assessing activity against both wild-type (WT) and doubly mutated (L858R/T790M, DM) forms of EGFR (Table 1).

**Table 1:** SAR for the variations in aniline and quinazoline substitution against EGFR WT and DM

No	Core	R	WT pIC_50_	DM pIC_50_
**4**			8.9 ± 0.05	8.5 ± 0.3
**7**			8.8 ± 0.1	8.4 ± 0.03
**8**			8.3 ± 0.07	8.0 ± 0.6
**9**			7.8 ± 0.2	7.5 ± 0.1
**10**			8.1 ± 0.09	7.9 ± 0.09
**11**			8.3 ± 0.1	7.7 ± 0.5
**12**			8.2 ± 0.04	8.4 ± 0.04
**13**			8.0 ± 0.05	7.8 ± 0.7
**14**			7.7 ± 0.00	6.2 ± 0.00
**15**			7.8 ± 0.03	7.3 ± 0.3

pIC_50_ values determined after 30 min incubations are a mean of at least 3 replicates.

All compounds demonstrated potent inhibition. For the acrylamides, varying the aniline substituent showed a variation in potency with the 2-fluoro-3,4-dichloro- (4) and 2-fluoro-3-chloro- (7) analogues being more potent than the 3-chloro-4-fluoro- (8) and 2-fluoro-5-chloro- (9) species against both WT and DM forms. Removal of the quinazoline 7-methoxy group showed a loss in potency, which was greater against DM than WT. As expected for anilinoquinazoline-based inhibitors^16,17^, the series as a whole showed increased potency for WT compared to the DM form. The differences in potency were far less significant for the propiolamides.

Variations in the cyclic amide spacer group bearing the acrylamide showed intriguing variations, changing the piperidine (8) to *R*-pyrrolidine 16 showed a drop in potency for both WT and DM EGFR but potency was regained in the *S*-enantiomer 17 (Table 2). The azetidine 18 showed increased DM potency relative to the piperidine. Again, these effects were absent or much less apparent in the propiolamides (19-21).

**Table 2:** SAR for the cyclic amine spacer group against EGFR WT and DM

No	Core	R	WT pIC_50_	DM pIC_50_
**8**			8.3 ± 0.07	8.0 ± 0.6
**16**			6.5 ± 0.08	7.0 ± 0.2
**17**			8.3 ± 0.2	8.2 ± 0.3
**18**			8.4 ± 0.05	8.8 ± 0.5
**11**			8.3 ± 0.1	7.7 ± 0.5
**19**			7.8 ± 0.09	8.2 ± 0.1
**20**			7.6 ± 0.05	7.8 ± 0.5
**21**			8.0 ± 0.1	8.0 ± 0.3

pIC_50_ values were determined after 30 min incubation are a mean of at least 3 replicates.

Glutathione reactivity analysis was carried out for matched pairs **16** and **19**, **17** and **20**, demonstrating the large differences in reactivity between the propiolamides and acrylamides (Table 3).

**Table 3:** GSH half-life of binding enantiomers **16** and **17,** **19** and **20** compared to osimertinib (**2**)

	*	Warhead	t_1/2_	t_1/2_ relative to osimertinib
2	Osimertinib		150 m	1
16	*R*	Acrylamide	36 h	14
17	*S*		41 h	16
19	*R*	Propargylamide	50 m	0.3
20	*S*		45 m	0.3

**Fig. 5:**
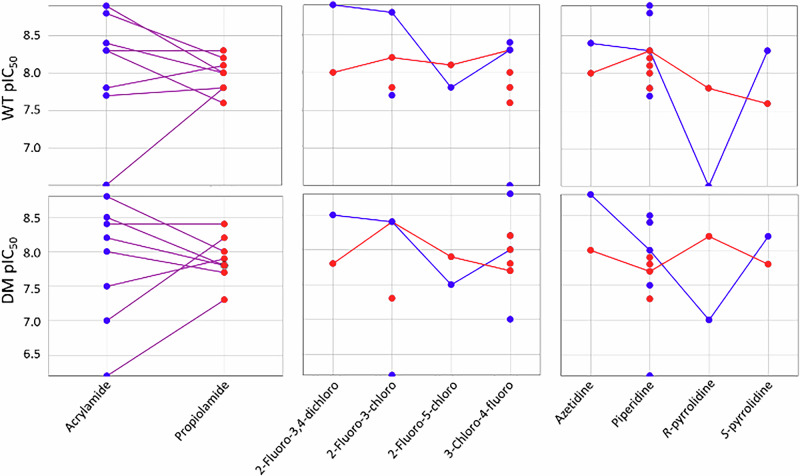
Matched molecular pair analysis of DM and WT EGFR potency comparing the warheads, aniline, and cyclic amine spacers. Blue denotes the acrylamides, and red denotes propiolamides, line connections show matched pairs.

Exploring these SAR relationships using matched pairs analysis emphasised the much broader range of activities with the acrylamide warhead (blue) than the propiolamide (red) (Fig. 5).

Given the much higher reactivity of propiolamides, this suggests that the activity for compounds containing this warhead is driven by the high reactivity and that changes in non-covalent affinity (at the selected incubation time) are masked when reactivity is too high. In contrast, the acrylamide warhead, with its lower reactivity, enables the elucidation of more nuanced SAR. This is apparent in the pairwise analysis of the aniline substituent for which the 2-fluoro-3,4-dichloro- and 2-fluoro-3-chloro- derivatives are more potent in the acrylamides (both WT and DM) whereas the propiolamides are much closer to each other in activity. Likewise, for the cyclic amine linkers, a clear preference for azetidine, piperidine, and *S*-pyrrolidine compared to the *R*-enantiomer can be clearly seen in the acrylamides but not the propiolamides.

### Differences in activity between enantiomers

**Fig. 6:**
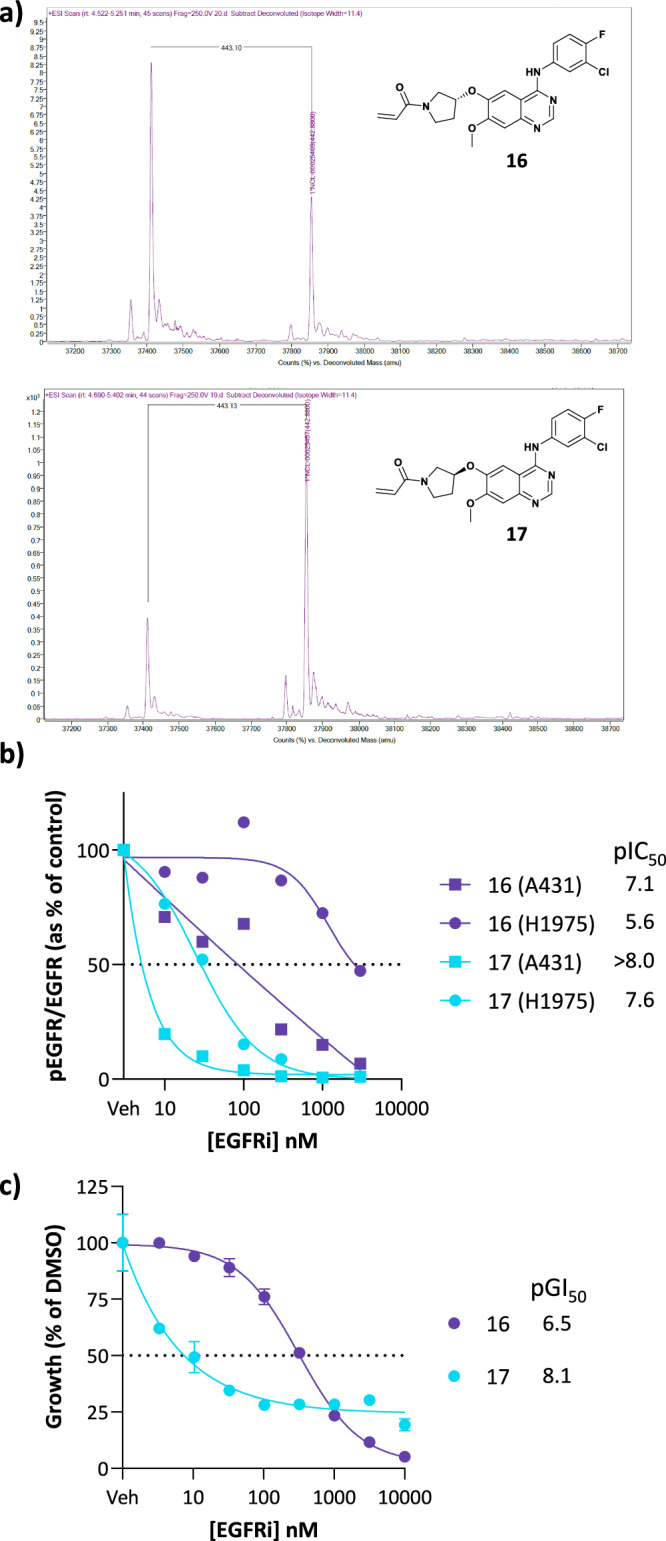
Characterisation of 16 and 17 by mass spectrometry and cellular pharmacology. **a** Intact protein mass spectrometry analysis comparing the degree of modification between 16 and 17; (**b**) Cellular pEGFR inhibition determined by HTRF in A431 and H1975 cells (*n* = 1); (**c**) Concentration response curves of the A431 cell viability in response to inhibitors (72 h) (*n* = 1, error = SD).

Intact protein mass spectrometry analysis was carried out on all acrylamide analogues (see SI) and pleasingly, all demonstrated formation of a covalent adduct with WT EGFR with a single binding event. Around a two-fold increase in the level of protein modification by *S-*pyrrolidine 17 compared to the less potent *R*-enantiomer 16 was observed (Fig. 6a), which is consistent with the measured EGFR potencies in the TR FRET assay.

In cellular EGFR phosphorylation (Fig. 6b), the more potent *S*-enantiomer 17 showed increased inhibition by more than an order of magnitude (pIC_50_ > 8.0 compared to 7.1) in WT EGFR overexpressing A431 cell lines. The difference in potency was also apparent in DM expressing H1975 cells. This difference further translated into growth effects in A431 cells, with 17 demonstrating a pGI_50_ of 8.1 compared to 6.5 for less potent *R*-enantiomer 16 (Fig. 6c).

**Fig. 7:**
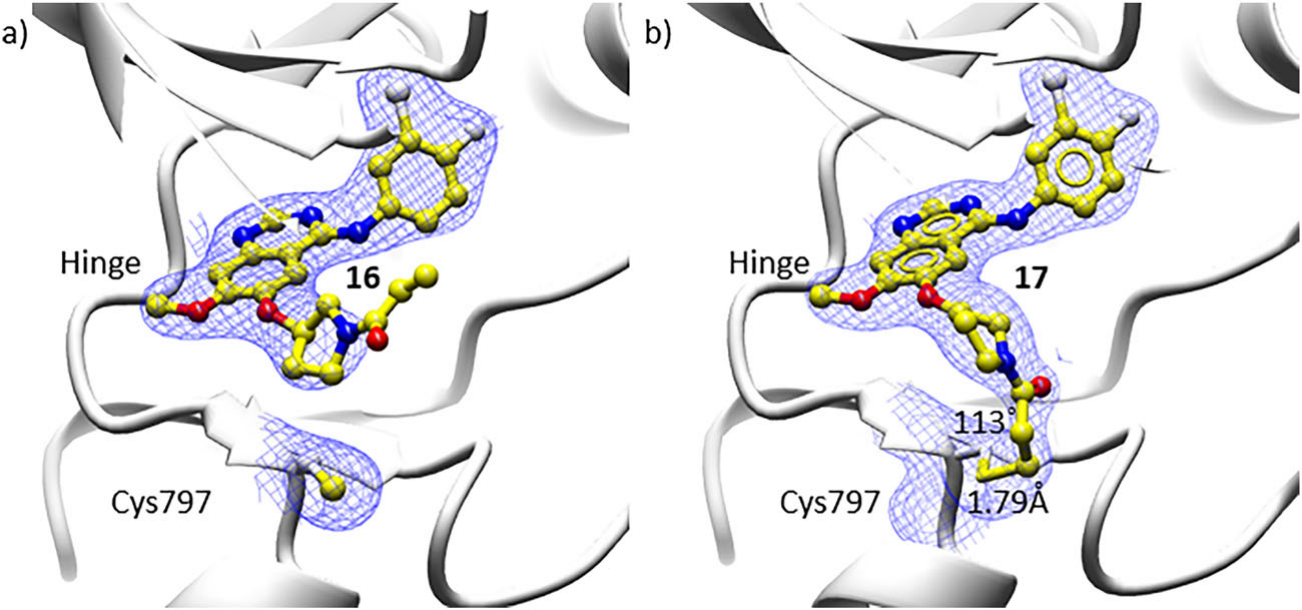
X-ray crystal structures of 16 and 17 in complex with WT EGFR. **a** 16 (R-enantiomer) and **b** 17 (Senantiomer). Continuous electron density 2Fo-Fc refinement map (blue mesh contoured to contoured to 0.15e/Å3 (1.0 r.m.s.d)) indicating the presence of a covalent bond with Cys797 (bond length 1.79Å, bond angle 113˚) for 17 but not 16. The difference map (Fo-Fc) with 17 omitted for refinement showing clear evidence of adduct formation (Fig. S4). Secondary structure shown in white ribbon, and electrostatic surface shown for active site (PDB: 9FZS 16, 9FZR 17).

X-ray crystallography was utilised to understand the structural causes of the significant differences in activity. Crystal soaking was used to obtain X-ray crystal structures of 16 and 17 bound to WT EGFR. The crystal structures clearly showed the formation of a covalent bond in the potent *S*-enantiomer 17, which was not observed for the *R*-enantiomer 16 (Fig. 7). The carbon-sulphur bond between 17 and Cys797 in WT EGFR demonstrates a bond length of 1.79 Å and bond angle 113˚. These measurements align with standard sulphur-carbon bonds and, therefore suggest the formation of an unstrained covalent bond.

To further assess the impact of the chiral centre on covalency, kinetic characterisation for binding of 16 and 17 against WT EGFR was carried out (Table 4). This showed the same difference in binding affinity with more potent 17 showing a 6-fold lower *K*_I_ and 3-fold faster *k*_inact_ relative to 16. The kinetic fit for 16 is indicative of covalent bond formation consistent with the mass spectrometry despite not observing a covalent complex by X-ray crystallography.

**Table 4:** Kinetic characterisation of binding of enantiomers 16 and 17 to WT EGFR

	*K*_I_ / nM	*k*_inact_ / 10^3^ s^−1^	*k*_inact_/*K*_I_ / 10^6 ^M^−1^s^−1^
**16**	1.60 ± 0.05	1.3 ± 0.03	0.82 ± 0.010
**17**	0.26 ± 0.02	3.8 ± 0.27	15 ± 0.021

The difference in propensity for covalent binding between 16 and 17 almost certainly arises because the stereochemical configuration of 16 disfavours the protein-bound conformation required to form a covalent bond. It is interesting that this manifests in a difference in both *K*_I_ and *k*_inact_. This would not be the case if it was dependent on reactivity alone. The observation suggests that the formation of the covalent bond starts before the non-covalent interactions are complete along the binding coordinate and that the processes giving rise to *K*_I_ and *k*_inact_ are not fully independent of one another.

To further unpick the covalent and non-covalent contributions to binding, the potency of non-covalent acyl analogues of 16 (22) and 17 (23) was measured against EGFR WT and DM (Table 4). Whilst 22 shows comparable potency to 16, suggesting potency for this scaffold is driven by the non-covalent interaction, a loss of potency is seen for 23 compared to 17, confirming that the improved potency is a consequence of the covalent binding event. Table 5

**Table 5:** Potency of non-covalent analogues of **16** and **17** against EGFR WT and DM


	*	WT pIC_50_	DM pIC_50_
22	*R*	7.4 ± 0.16	7.6 ± 0.20
23	*S*	6.9 ± 0.11	7.5 ± 0.18

pIC_50_ values were determined after 30 min incubation are a mean of at least 3 replicates.

**Fig. 8:**
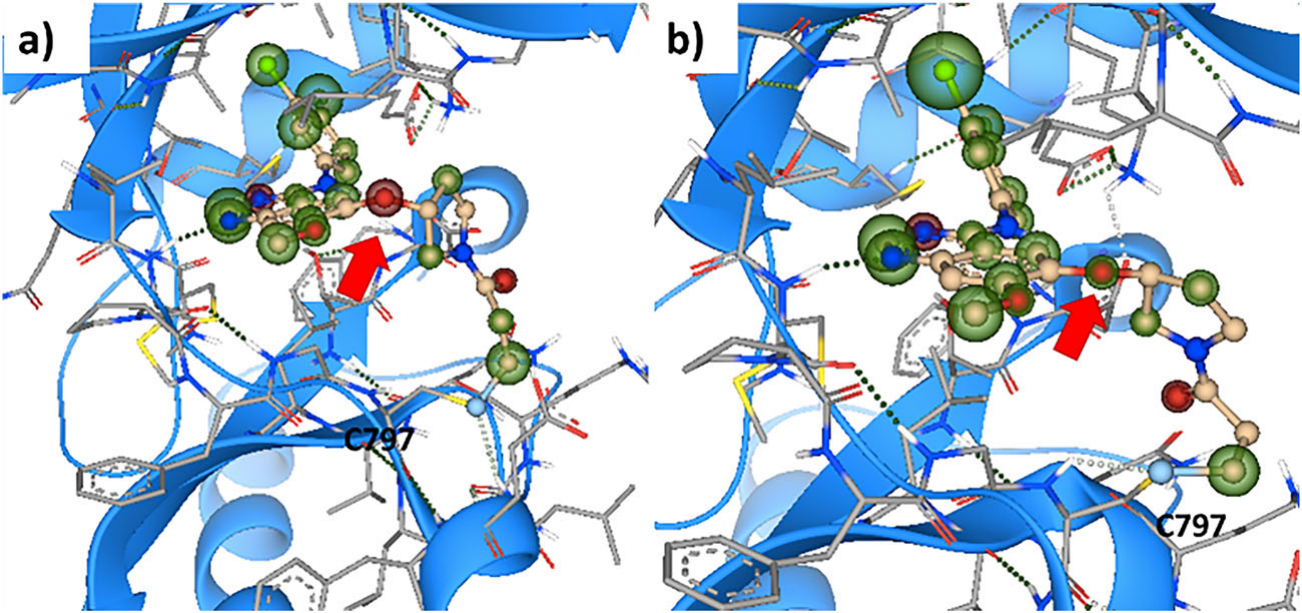
Covalent docking of 16 an 17 to EGFR. **a** 16 and **b** 17. Spheres around atoms denote their calculated affinity contributions (green – favourable, red – unfavourable).

Covalent docking using SeeSAR (v. 13.2) (https://www.biosolveit.de/SeeSAR) starting from the crystal structures of both compounds reproduced the bound crystal structure of 17 and gave estimated pIC_50_ values for 16 and 17 of 7.1 and 8.8 respectively (estimated by the HYDE^18^ scoring function), in reasonable agreement with experimental data. The docking study suggests the lower affinity of 16 arises from torsional strain surrounding the oxygen atom linking the quinazoline core with the pyrrolidine group (red arrow), which was observed for all adducts of 16 (Fig. 8a), but not in those of 17 (Fig. 8b).

Molecular dynamics simulations of EGFR-bound and unbound ligands in explicit water were carried out (Fig. S1 and S2). These indicated that the EGFR-bound conformation of 17 (Fig. S1a) was also the lowest energy unbound conformation, whilst for 16, the non-covalent bound conformation (Fig. S1b) seen in the crystal structure was significantly more favourable than the covalently bound conformation (Fig. S1c) generated in the docking studies.

Overall, energy minimisation calculations performed at QM and MM levels of theory and all-atom simulations of unbound 16 and 17 in explicit water indicate that the activity difference is likely driven by intrinsic conformational factors observed in the unbound ligand. This results in the preference for 17 to assume a conformation capable of forming a covalent adduct, whilst 16 needs to adopt a less favoured, strained conformation in order to form the adduct. This was validated further through metadynamic simulations. For 17, the conformation resembling that observed in the crystal structure is in the deep energy minimum, with another reactive conformation populating the adjacent minimum (Fig. S3a). For 16, the reactive conformation is far from the minimum, consistent with the strain observed in the molecular docking calculations, whilst local energy minima are populated with snapshots resembling the non-covalent bound conformation of 16 (Fig. S3b). The distribution of the data points of the scatter plots from the dihedral analysis (Fig. S2d–f) closely match the low energy area of the conformational space explored by the metadynamics (Fig. S3). This indicates that the major energy barrier for the transition of 16 conformation B to conformation C primarily exists in the process of dihedral twisting. Well-tempered metadynamics calculated the energy barrier between conformations B and C to be +16 kJmol^−1^. This fits well with the potency trend and provides a possible explanation for the significantly lower activity of 16 compared to 17.

## Conclusions

A fluorescent probe displacement assay for the EGFR active (ATP-binding) site was used as the primary assay to explore small differences in non-covalent affinity and for SAR analysis, including matched molecular pairs, for a set of poziotinib analogues. This revealed SAR trends for both the aniline and cyclic amine substructures that were much more readily apparent in the acrylamide than the propiolamde series. Presumably, this is because the potency of the propiolamides arises largely from their increased reactivity. However, in general, the propiolamides did not show higher potency as a class, which would be expected if the compounds simply all had greater *k*_inact_ values (we would expect *K*_I_ values to be similar for both across matched molecular pairs). The differences may be due in part to the less stringent geometries that are required for a propiolamide relative to an acrylamide for thiol addition, although it would be expected that this would be more apparent in the cyclic amine SAR than the aniline.

Surprisingly, the work identified a pair of enantiomers **16** and **17**, with significantly different activities. Intact protein mass spectrometry, X-ray crystallography, kinetic and computational analysis led us to conclude that the inversion of stereochemistry from **17** to **16** disfavours the reactive conformation able to form a covalent bond, such that the lower potencies and growth effects observed for **16** compared to **17** are caused by the lower level of covalent bond formation by **16**. This observation further emphasises the importance of geometry as well as reactivity in the design of covalent inhibitors.

This study demonstrates that a fluorescent probe displacement assay can be used as a primary assay in the optimisation of covalent inhibitors and in this case revealed SAR arising from geometrical constraints required for covalent reaction. The validity of the data is supported by its translation into cellular mechanistic and phenotypic effects for a pair of compounds that differ only in their chirality. The determination of *K*_I_ and *k*_inact_ values for the enantiomeric pair are consistent with the TR-FRET data, further validating it as a primary assay. The important condition for this is that in this case, compounds with similar levels of reactivity were compared. Other bioassay formats may be equally valid in this regard.

The work demonstrates that elucidation of non-covalent SAR and selectivity is clearer with less reactive warheads in this case. If transferable to future projects, it will be desirable to work with compounds with reactivity in the same range as those of drug-like acrylamides not only because these are established to deliver efficacy in vivo but also because the reduced reactivity provides clearer SAR. For projects starting with more reactive warheads, it would be desirable to modulate the reactivity into this range at an early stage.

## Methods

### GSH reactivity

Compounds were suspended to 10 mM in 100% DMSO and were added to 4.6 mM GSH to achieve the final compound concentration of 50 µM in pH 7.4 phosphate buffer before incubating at 37 °C. Compound reaction with GSH was monitored by LC-MS on a Waters Acquity UPLC system with PDA and ELSD operating in positive and negative ion electrospray mode, employing an Acquity UPLC BEH C18, 1.7 mm, 2.1 × 50 mm column with 0.1% formic acid and water-acetonitrile (5–95%) for gradient elution over 4 minutes.

### Protein Productions

EGFR constructs (Wild type, Triple mutant – C797S, L858R, T790M) were produced using the MultiBac system in SF9 cells grown at double density for 72 h. The cells were collected by centrifuging at 4000 × g for 10 min. Pellets were flash-frozen in liquid nitrogen and stored at –70 °C. Buffer A (25 mM Tris-HCl pH 8, 250 mM NaCl, 10% glycerol, 10 mM β-mercaptoEtOH) was supplemented with 5 mM MgCl_2_, 20 mM imidazole, EDTA-free protease inhibitor cocktail (Roche), 0.1 mg/ml DNase, 0.1 mg/ml RNase for lysis. The pellet was unfrozen and resuspended in lysis buffer, sonicated for 5 min at 30% 5 s on, 20 s off, and centrifuged at 100,000 *g* for 30 min. The supernatant was filtered through a 0.45 μm filter. 5 ml of Ni-NTA resin was added to the clarified lysate and incubated for 1 h at 4 °C. The resin was rinsed with wash buffer (buffer A with 20 mM imidazole), and the protein was eluted with buffer A with 300 mM imidazole. For ion exchange, the protein was dialysed into a buffer with 25 mM NaCl. Protein was loaded onto Q FF column and eluted with 1 M buffer using a gradient.

### Intact protein mass spectrometry

Compounds were suspended to 10 mM in 100% DMSO and were added to 4 µM EGFR to achieve the final compound concentration of 15 µM in 1% DMSO, in 25 mM TRIS pH8, 150 mM NaCL, 5% glycerol before incubating at ambient temperature for the 4 hr time prior to analysis. Following incubation, intact protein masses were determined using an Agilent 6530 Accurate Mass dual AJS/ESI Q-TOF instrument coupled to an Agilent 1260 Infinity II LC system. 1 µL of purified protein (~1 mg/mL) was injected onto a MS Pac DS-10 Desalter cartridge ((Thermo Fisher Scientific), PN: 089170, 2.1 × 10 mm) for desalting and reversed phase separation at 70 °C. The mobile phase was 0.1% (v/v) formic acid in LC-MS grade water (A) and LC-MS grade acetonitrile (B) with separation performed over 7.5 min. Sample desalting was achieved at 30% B for 2 min at 1 mL/min before reducing the flow rate to 0.2 mL/min for 2 min. Protein elution was achieved at 100% B for 0.5 min and 1 ml/min before re-equilibration at 30% for 1 min. Proteins were detected in positive ion mode using electrospray ionisation with nebuliser pressure of 45 psig, drying gas flow of 5 L/min, and source gas temperature of 325 °C. Sheath gas temperature of 400 °C and gas flow of 11 L/min, capillary voltage of 3500 V and nozzle voltage of 2000V were also used. Mass spectra were acquired using MassHunter Acquisition software (version B.08.00) over a mass range of 100–3000 m/z, at a rate of 1 spectra/s and 1000 ms/spectrum in standard mass range (3200 m/z) at 2 GHz. The instrument had been calibrated over the selected mass range prior to analysis.

### In-vitro TR-FRET analysis

Compounds (dissolved to 10 mM in DMSO) were dispensed into black low-volume 384 well assay plates (Corning) over a final concentration range of 100000, 30,000, 10,000, 3000, 1000, 300, 100, 30, 10, and 3 nM using an Echo 550 (Labcyte). Positive control compound and DMSO as a negative control were dispensed into the first and last well, respectively. Each well was backfilled with DMSO to a final volume of 200 nl, resulting in final assay DMSO concentrations of 1%. 19.8 μl of premixed solution containing final assay concentration of CtermHisTag-mEGFR (Wild type) (1.25 nM), Probe (N-(4-(4-(3-((4-((3-chloro-4-fluorophenyl)amino)-7-methoxyquinazolin-6-yl)oxy)propyl)piperazin-1-yl)-4-oxobutyl)-3-(5,5-difluoro-7,9-dimethyl-5H-5l4,6l4-dipyrrolo[1,2-c:2’,1’-f ][1,3,2]diazaborinin-3-yl)propenamide) (100 nM), and Tb-anti-His Antibody 61HI2TLF (Cisbio Assay) in a buffer containing 20 mM Tris pH 7.5, 100 mM NaCl, 100 μg/ml bovine serum albumin, was added to each well and incubated with shaking at room temperature for 30 mins. Plates were read using a PheraStar FS (BMG Labtech) at Ex337nm Ex490/520 nm. The data were analysed using GraphPad Prism/Dotmatics Studies Software. Assays were conducted in technical replicate and repeated as a biological duplicate.

### Structure determination

Crystallization was performed at 20 °C using the sitting drop vapour diffusion method dispensed using Mosquito (TTP labtech). Purified Wild-type EGFR was produced as described by Yun et al.^19^. Crystals of Wild-type EGFR were grown using EGFR (10 mg/ml), 0.1 M HEPES pH7.4, 40%PEG400. Crystals were soaked with compounds **16** and **17** overnight before being flash-frozen in liquid nitrogen before data collection. X-ray diffraction data were recorded at the i04 beamline, Diamond Light Source (Oxford, UK). Data processing was carried out using DIALS^20^, POINTLESS/AIMLESS^21^ and other CCP4 programmes^22^ run within the CCP4i2 GUI. Phasing by molecular replacement using pdb 2ITZ as a search model was carried out using PHASER^23^. Iterative rounds of model building and refinement were performed using COOT^24^ and REFMAC5^25^, respectively. Figures were prepared using CCP4MG^26^. Crystallographic data may be found in Supplementary Table 1.

### Cell assays

#### HTRF method (A431)

Cells were plated at 20,000 cells per well in 96 well plates and placed at 37°C 5% CO_2_. Once adhered (after 24 h) cells were treated with compounds dissolved in DMSO at a final concentration of 0.1% DMSO in media. Compounds were diluted in media then added to cells for 2.5 hours in duplicate. 100 ng/ml EGF (ThermoFisher, PHG0311) was is added to all compound treated cells as well as a control for 30 mins. Compound and media were removed from the cells, then 50ul of HTRF lysis buffer was added (lysis buffer was diluted from 4X stock to 1X in DI water, with 1% blocking agent also added). Cells were lysed on a plate shaker (2000 rpm) for 30 minutes at room temperature. pEGFR expression was monitored using the Cisbio Phospho-EGFR (Tyr1068) cellular kit (64EG1PEH). Total EGFR expression was monitored using the Cisbio Total EGFR cellular kit (64NG1PEH) as per manufacturer’s instructions. Fluorescence emission was read at two different wavelengths (665 nm and 620 nm) on a PheraStar. Results were calculated as the ratio of pEGFR/ total EGFR and then the percentage of 0 µM control. Data is *n* = 1 from 2 replicates.

#### Growth inhibition in adherent cell lines

A431 cells purchased from ECACC, Cat. 85090402, were plated on day 0 in a 96-well plate at a density known to allow for exponential growth over 72 h in DMEM (Sigma-Aldrich, Cat No. D5796) supplemented with 10% FBS (Gibco, Cat No. 10270-106). On day 1, the compounds were diluted to the required concentration in DMEM + 10% FBS media, ensuring that the final DMSO concentration was 0.1% once added to cells. The cells were then incubated for 72 h at 37 °C with 5% CO_2_. Cells were then fixed by adding 50% (wt/vol) TCA to each well of the plate and left at 4 °C for 1 h. The plates were then washed thoroughly with water, 100 μL of 0.4% SRB solution was added to the wells, and left at room temperature for 30 min. The plates were rinsed with 1% AcOH and then left to air-dry in a drying cabinet for 1 h. Once dry, 100 μL of 10 mM tris pH 10.5 was added to each well and the plates were placed on a plate shaker for 10 min. The absorbance was read at 570 nm using a FluoStar Omega plate reader. Data is *n* = 1 from 3 replicates,

#### Kinetic analysis

Measurement of *K*_I_, *k*_inact_ and *K*_I_/*k*_inact_ was carried out by AssayQuant® Technologies inc.

## Supplementary information

Supplementary material

## Data Availability

Experimental details of compound synthesis and characterisation, protein mass spectrometry analysis computational methods and X-ray crystallography are provided in the Supplementary Information. X-ray crystallographic data are available in the Protein Data Bank (codes: 9FZS for 16 and 9FZR for 17).
