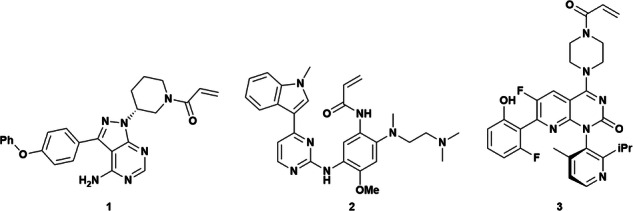# Author Correction: Factors affecting irreversible inhibition of EGFR and influence of chirality on covalent binding

**DOI:** 10.1038/s42004-025-01551-w

**Published:** 2025-05-19

**Authors:** Pasquale A. Morese, Ayaz Ahmad, Mathew P. Martin, Richard A. Noble, Sara Pintar, Lan Z. Wang, Shangze Xu, Andrew Lister, Richard A. Ward, Agnieszka K. Bronowska, Martin E. M. Noble, Hannah L. Stewart, Michael J. Waring

**Affiliations:** 1 Cancer Research Horizons Newcastle Drug Discovery Unit, Chemistry, School of Natural and Environmental Sciences, Bedson Building, Newcastle University, Newcastle upon Tyne, UK; 2 Chemistry, School of Natural and Environmental Sciences, Bedson Building, Newcastle University, Newcastle upon Tyne, UK; 3 Cancer Research Horizons Newcastle Drug Discovery Unit, Translational and Clinical Research Institute, Paul O’Gorman Building, Newcastle University, Newcastle upon Tyne, UK; 4 Oncology iMed, R&D, AstraZeneca, Cambridge, UK

**Keywords:** Drug discovery and development, X-ray crystallography, Bioconjugate chemistry, Mechanism of action

Correction to: *Communications Chemistry* 10.1038/s42004-025-01501-6, published online 09 April 2025

In the version of the article initially published, in Fig. 1, a methoxy group was missing from structure 2 and has now been amended in the HTML and PDF versions of the article, as seen in Fig. 1 below.

Fig. 1 Original and corrected Fig. 1

Original Fig. 1
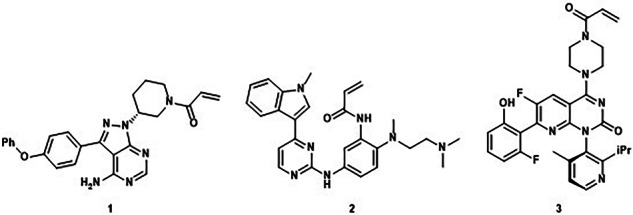


Corrected Fig. 1