# Supplementary material for: Factors affecting irreversible inhibition of EGFR and influence of chirality on covalent binding
Source: Commun Chem. 2025 Apr 9;8:111. doi: 10.1038/s42004-025-01501-6 (PMC11982232; doi:10.1038/s42004-025-01501-6)
Supplement: Supplementary file 1 — Supplementary material [file 42004_2025_1501_MOESM1_ESM.pdf]

Supporting Information

Factors affecting irreversible inhibition of EGFR and influence of chirality on covalent binding

Pasquale A. Morese,[a] Ayaz Ahmad,[b] Mathew P. Martin,[c] Richard A. Noble,[c] Sara Pintar,[c] Lan Z. Wang,[c] Shangze Xu,[b] Andrew Lister,[d] Richard A. Ward,[d] Agnieszka K. Bronowska,[b] Martin E. M. Noble,[c] Hannah L. Stewart,*[a] and Michael J. Waring*[a]

[a]Cancer Research Horizons Newcastle Drug Discovery Unit, Chemistry, School of Natural and Environmental Sciences, Bedson Building, Newcastle University, Newcastle upon Tyne, NE1 7RU, UK.
[b]Chemistry, School of Natural and Environmental Sciences, Bedson Building, Newcastle University, Newcastle upon Tyne, NE1 7RU, UK.
[c]Cancer Research Horizons Newcastle Drug Discovery Unit, Translational and Clinical Research Institute, Paul O'Gorman Building, Newcastle University, Newcastle upon Tyne, NE2 4HH, UK.
[d]Oncology iMed, R&D, AstraZeneca, Cambridge, CB4 0WG, UK.

Corresponding author email: mike.waring@ncl.ac.uk

Contents

Supplementary Figures 2
General procedures 5
NMR and HPLC Spectra for key compounds 22
Intact Protein Mass Spectrum 29
Computational Methods 33
Crystallographic Table 34
References 34

Supplementary Figures

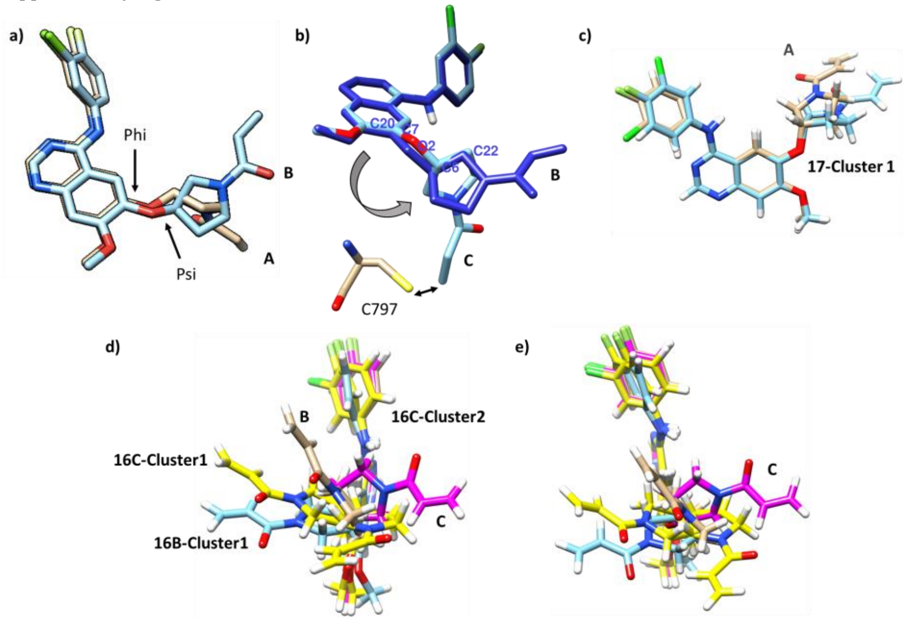

Figure S1: a) Comparison of the crystal bound conformations of **17** (A – beige) and 16 (B – pale blue); b) comparison of the covalent and non-covalent conformations of **16**: non-covalent crystal bound (B – blue) and covalently docked (C – pale blue); c) comparison of conformation A of **17** with the highest-populated cluster generated through molecular dynamics trajectories starting from conformation A; d) comparison of conformations B and C of **16** with the highest-populated clusters generated through molecular dynamics trajectories starting from conformation B; e) comparison of conformations B and C of **16** with the highest-populated clusters generated through molecular dynamics trajectories starting from conformation C.

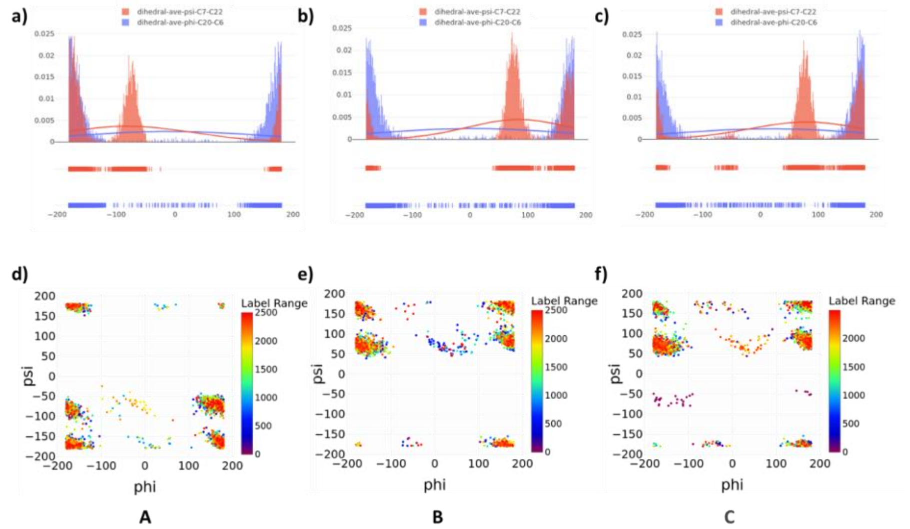

Figure S2: a-c) distribution of phi and psi dihedral angles in conformations A (a), B (b) and C (c) during molecular dynamics simulation. The plots show different preferences in psi angle (red) between poziotinib isomers **17** and **16**; d-f) scatter plots of phi and psi dihedrals from the MD simulation trajectories. The data points, which correspond to the dihedral values in MD snapshots, are coloured by the frame index (purple – start of the simulation, following through blue, green, yellow and red – end of the simulation).

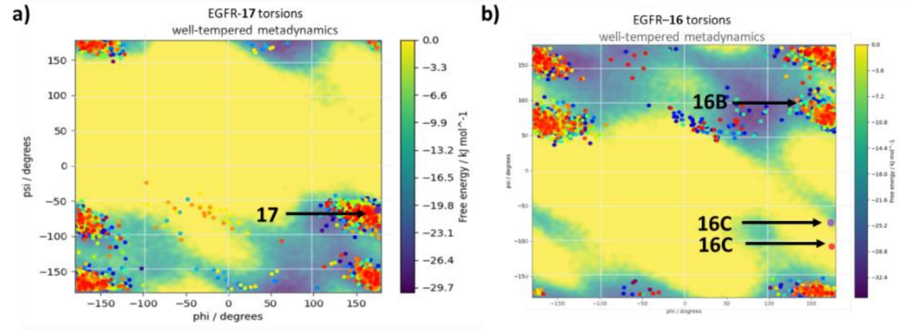

Figure S3: Combination of 3 individual replicas of our metadynamics simulations overlayed with 2,500 individual frames of the phi and psi dihedrals displayed as a scatter plots for a) **17,** showing the conformation resembling that seen in the crystal structure (A) in a deep energy minimum, with another reactive conformation populating the adjacent minimum; b) **16**, showing that the reactive conformation (C) is far from the minimum, which is consistent with the strain observed in the molecular docking calculations. Local energy minima are populated with snapshots resembling of conformation B.

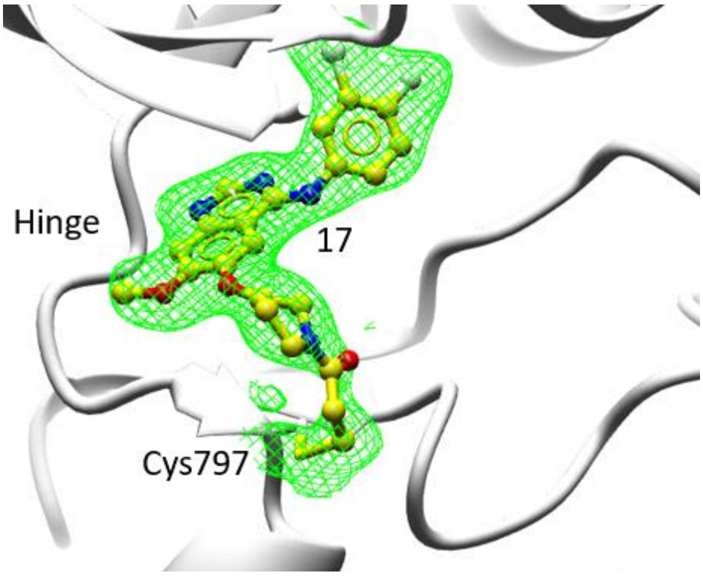

Figure S4: Crystal structure of WT EGFR bound to **17,** showing **17** bound at the hinge and engaging Cys797 with a covalent bond. Show in green mesh is the difference map (Fo-Fc)(contoured to 0.15e/Å^3 (2.7r.m.s.d)) with **17** omitted for refinement showing clear evidence of adduct formation.

General procedures

Chemicals and solvents
All commercial reagents were purchased from reputable chemical companies. The chemicals were of the highest available purity. Unless otherwise stated, chemicals were used as supplied without further purification. Anhydrous solvents were obtained from either Aldrich or Acros and were stored under nitrogen. Petrol refers to the fraction with a boiling point between 40 and 60 °C.

Chromatography
TLC utilised to monitor reaction progress was conducted on plates pre-coated with silica gel (Merck 60F_{254}). The eluent was as stated (where this consisted of more than one solvent, the ratio is stated as volume:volume) and visualisation was either by short wave (254 nm) ultraviolet light. 'Flash' MPLC was carried out on a pre-paced silica columns. Semipreparative HPLC was carried out on an Agilent instrument passing through a Waters XSelect column employing a C_{18} 19 × 150 nm, 3.5 Å column (eluent: (acidic) 0.1% formic acid (aq)/MeCN, (basic) 0.1% NH_3(aq)/MeCN), using a UV detector at 254 nm and a flow rate of 20 mL/min.

Analytical techniques
Melting points were determined using a VWR Stuart SMP40 apparatus and are uncorrected. Optical rotations were recorded on a PolAAr 3001 Automatic Polarimeter (Optical Activity Ltd, Cambridgeshire, UK), units of $[\alpha]_D$ are given in 10^{-1} deg cm^2 g^{-1}. LC-MS was carried out on a Waters Acquity UPLC system with PDA and ELSD operating in positive and negative ion electrospray mode, employing an Acquity UPLC BEH C18, 1.7 mm, 2.1 × 50 mm column with 0.1% formic acid and water-acetonitrile (5–95%) for gradient elution. FTIR spectra were recorded on a Agilent Cary 630 FTIR Spectrometer as a neat sample. Bond stretch frequencies are reported as br (broad), s (sharp), m (medium) or w (weak) based on their relative intensities. UV spectra were obtained using a U-2001 Hitachi Spectrophotometer with the sample dissolved in ethanol. ^{1}H, ^{13}C, ^{15}N, ^{19}F nuclear magnetic resonance (NMR) spectra were obtained as either $CDCl_3$, CD_3OD or DMSO-d_6 solutions and recorded at 500 MHz, 126 MHz, 700 MHz and 470 MHz respectively, on either a Bruker Avance III 500 or 700 spectrometer. Chemical shifts are quoted in parts per million (δ) referenced to the appropriate deuterated solvent employed. Multiplicities are indicated by s (singlet), d (doublet), t (triplet), q (quartet), p (pentet), m (multiplet), br (broad) or combinations thereof. Coupling constant values are given in Hz. Homonuclear and heteronuclear two dimensional NMR experiments were used where appropriate to facilitate assignment of chemical shifts. The numbering system used in the assignment of aromatic carbons and hydrogens are done so according to IUPAC nomenclature. All final compounds are >95% pure by HPLC analysis.

***tert*-butyl 4-(3-bromopropyl)piperazine-1-carboxylate**

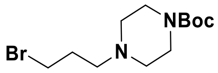

To a stirred solution of 1-Boc-piperzine (1.00 g, 5.37 mmol) in dichloromethane (15 mL) at 0°C was added triethylamine (596 mg, 5.90 mmol) and 1,3-dibromopropane (0.602 mL, 5.90 mmol). The reaction mixture was warmed to room temperature over 18 h. The reaction mixture was diluted with dichloromethane (30mL) and washed with saturated aqueous sodium hydrogen carbonate solution (20 mL), water (10 mL) and brine (10 mL), dried (Na_2SO_4) and concentrated under reduced pressure. The crude material was purified by flash column chromatography eluting with ethyl acetate (30%) in 40-60 petroleum ether to yield the title compound as an oil (70%). ^{1}H NMR (500 MHz, Chloroform-d) δ 3.49 (t, J = 6.6 Hz, 2H), 3.44 (t, J = 5.2 Hz, 4H), 2.50 (t, J = 6.9 Hz, 2H), 2.40 (t, J = 5.1 Hz, 4H), 2.06 – 2.01 (m, 2H), 1.48 (s, 9H); ^{13}C NMR (126 MHz, Chloroform-d) δ 154.8, 79.7, 56.5, 53.1, 43.5, 31.7, 29.9, 28.4

Data is consistent with what has been reported in the literature.[1]

tert-butyl 4-(3-((4-((3-chloro-4-fluorophenyl)amino)-7-methoxyquinazolin-6-yl)oxy)propyl)piperazine-1-carboxylate

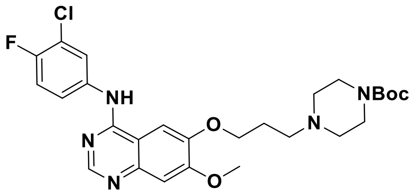

To a stirred solution of 4-((3-chloro-4-fluorophenyl)amino)-7-methoxyquinazolin-6-ol (300 mg, 0.941 mmol) in DMF (3.0 mL) was added potassium carbonate (259 mg, 1.88 mmol) followed by *tert*-butyl 4-(3-bromopropyl)piperazine-1-carboxylate (346 mg, 1.12 mmol). The reaction mixture was heated at 80°C for 5 h then concentrated Under reduced pressure. The residue was dissolved in dichloromethane (50 mL) and washed with brine (10 mL), dried (Na_2SO_4) and concentrated under reduced pressure. The crude material was purified by flash column chromatography to yield the title compound (49%). ^{1}H NMR (500 MHz, Chloroform-d) δ 8.65 (s, 1H), 7.82 (dd, J = 2.7, 6.5 Hz, 1H), 7.68 (s, 1H), 7.53 (ddd, J = 2.7, 4.1, 8.9 Hz, 1H), 7.22 (s, 1H), 7.19 (s, 1H), 7.13 (t, J = 8.7 Hz, 1H), 4.13 (t, J = 6.6 Hz, 2H), 3.96 (s, 3H), 3.46 – 3.37 (m, 4H), 2.53 (t, J = 7.0 Hz), 2.38 (t, J = 5.1 Hz, 4H), 2.06 (dd, J = 6.3, 13.1 Hz, 2H), 1.44 (s, 9H); ^{13}C NMR (126 MHz, Chloroform-d) δ 156.2, 155.7 (d, $^2J_{C-F}$=247.2 Hz), 155.2, 154.8, 153.4, 149.1, 147.5, 135.4, 124.2, 121.8 (d,$^4J_{C-F}$=6.6 Hz), 120.9, 116.7 (d,$^3J_{C-F}$=20.8 Hz),108.9, 107.9, 100.6, 79.9, 67.5, 56.2, 54.9, 53.0, 44.0, 28.4, 26.4; LRMS (ES+) m/z = 547 [M+H]+.

Data is consistent with what has been reported in the literature.[2]

_N_-(3-chloro-4-fluorophenyl)-7-methoxy-6-(3-(piperazin-1-yl)propoxy)quinazolin-4-amine

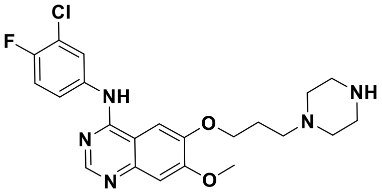

Tert-butyl4-(3-((4-((3-chloro-4-fluorophenyl)amino)-7-methoxyquinazolin-6-yl)oxy)propyl)piperazine-1-carboxylate (234 mg, 0.422 mmol) in trifluoroacetic acid (1 mL) and dichloromethane (1 mL) was stirred at room tempertaure for 3 h. The reaction mixture was diluted with dichloromethane (20 mL), basified to pH 10, and extracted with dichloromethane (3 x 20 mL). The combined organic layers were dried (Na_2SO_4) and concentrated under reduced pressure to yield the title compound as yellow solid (180 mg, 91%). R_f : 0.56 (EtOAc:MeOH:Conc. Aq Ammonia: 9:1:0.1); m.p. 115ºC-116 ºC ; ^{1}H NMR (500 MHz, Chloroform-d) δ 8.66 (s, 1H), 7.91 (dd, J = 6.5, 2.6 Hz, 1H), 7.55 (ddd, J = 8.4, 3.9, 2.5 Hz, 1H), 7.45 (s, 1H), 7.25 (s, 1H), 7.19 – 7.13 (m, 2H), 4.19 (t, J = 6.6 Hz, 2H), 4.00 (d, J = 1.3 Hz, 3H), 2.93 (t, J = 4.8 Hz, 4H), 2.59 (t, J = 7.0 Hz, 2H), 2.55 – 2.44 (m, 4H), 2.12 (q, J = 7.0 Hz, 2H); ^{13}C NMR (126 MHz, DMSO-d$_6$) δ 156.5, 155.0, 154.6 (d $^2J_{C-F}$ =247.2 Hz) 153.1, 148.9, 147.4, 137.3,122.8 (d,$^4J_{C-F}$ =6.6 Hz),119.3 (d, J_{C-F} =16.9 Hz), 117.04 (d, J_{C-F} =21 Hz),109.3, 107.8, 103.0, 67.7, 56.3, 55.7, 54.7, 46.0, 26.5.

Methyl 4-azidobutanoate

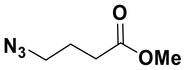

To a stirred solution of methyl 4-chlorobutyrate (500 mg, 3.66 mmol) in DMSO (3.0 mL) was added sodium azide (333 mg, 5.10 mmol). The reaction mixture was heated at 45°C for 24 h then cooled down to room temperature and quenched with water (10 mL). The aqueous layer was extracted with diethyl ether (3 x 30 mL). The combined organic layers were dried (Na_2SO_4) and concentrated under reduced pressure to yield the title compound as a

colourless oil (95%). ^{1}H NMR (500 MHz, Chloroform-d) δ 3.70 (d, J= 3.0 Hz, 3H), 3.37 (td, J= 6.7, 2.7 Hz, 2H), 2.44 (td, J= 7.3, 2.7 Hz, 2H), 1.93 (pd, J= 7.1, 2.9 Hz, 2H).

Data is consistent with what has been reported in the literature.[3]

4-azidobutanoic acid

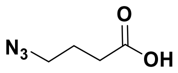

Methyl 4-azidobutanoate (500 mg, 3.49 mmol) was dissolved in 2M aqueous sodium hydroxide solution (6.99 mL), a few drops of methanol were added to the reaction and it was stirred at room temperature overnight. The reaction mixture was concentrated under reduced pressure to remove the methanol. The aqueous layer was washed with diethyl ether (3 x 20 mL), acidified with 6M HCl to pH 1 and then extracted with diethyl ether (3 x 35 mL). The combined organic layers were dried ($MgSO_4$) and concentrated under reduced pressure to yield the title compound as colourless oil (98%). ^{1}H NMR (500 MHz, Chloroform-*d*) δ 3.40 (t, *J*= 6.7 Hz, 2H), 2.49 (t, *J*= 7.2 Hz, 2H), 1.93 (p, *J*= 6.9 Hz, 2H).^{13}C NMR (126 MHz, Chloroform-*d*) δ 178.81, 50.47, 30.91, 23.95

Data is consistent with what has been reported in the literature.[4]

4-azido-1-(4-(3-((4-((3-chloro-4-fluorophenyl)amino)-7-methoxyquinazolin-6-yl)oxy)propyl)piperazin-1-yl)butan-1-one

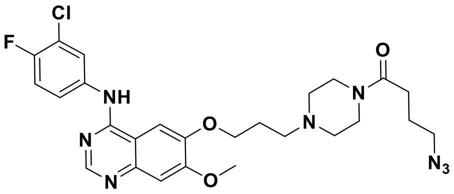

To a stirred solution of 4-azidobutanoic acid (16.0 mg, 0.123 mmol) in dichloromethane (0.6 mL) was added N,N-diisoporpyl-N-ethylamine (43.4 mg, 0.336 mmol) followed by HATU (63.0 mg, 0.168 mmol). The reaction mixture stirred at rt for 10 minutes, *N*-(3-chloro-4-fluorophenyl)-7-methoxy-6-(3-(piperazin-1-yl)propoxy)quinazolin-4-amine (50.0 mg, 0.110 mmol) was added and the reaction mixture stirred at room temperature for 18 h. The reaction was diluted with dichloromethane (25 mL), washed with water (7 mL), dried ($MgSO_4$) and concentrated under reduced pressure. The crude material was purified by flash column chromatography using SiO_2 NH_2, eluting with ethyl acetate to yield the title compound as white solid (40.0 mg, 65%). R_f 0.3 (10% MeOH:DCM); m.p. 177°C; IR: ν_{max}/cm^{-1} 2094 (s, N=N=N); UV λ_{max} (nm) 331,249,225; ^{1}H NMR (500 MHz, Chloroform-d) δ 8.65 (s, 1H), 7.82 (dd, J = 6.5, 2.7 Hz, 1H), 7.61 (s, 1H), 7.53 (ddd, J = 8.9, 4.1, 2.7 Hz, 1H), 7.23 (s, 1H), 7.17 (s, 1H), 7.14 (t, J = 8.8 Hz, 1H), 4.14 (t, J = 6.5 Hz, 2H), 3.97 (s, 3H), 3.61 (t, J = 5.0 Hz, 2H), 3.47 (dt, J = 6.5, 3.9 Hz, 2H), 3.36 (t, J = 6.4 Hz, 2H), 2.56 (t, J = 7.0 Hz, 2H), 2.48 – 2.40 (m, 4H), 2.38 (d, J = 7.2 Hz, 2H), 2.08 (q, J = 6.8 Hz, 2H), 1.90 (p, J = 6.8 Hz, 2H); ^{13}C NMR (126 MHz, Chloroform-d) δ 170.38, 156.31, 155.87, 155.38, 153.56, 149.23, 147.70, 135.49, 124.25, 121.93 – 121.77, 121.30 – 120.98 , 116.94 – 116.56 ,109.05, 108.11, 100.85, 67.63, 56.34, 54.89, 53.53, 52.95, 51.07, 45.54, 41.76, 29.83, 26.54, 24.57.

4-amino-1-(4-(3-((4-((3-chloro-4-fluorophenyl)amino)-7-methoxyquinazolin-6-yl)oxy)propyl)piperazin-1-yl)butan-1-one

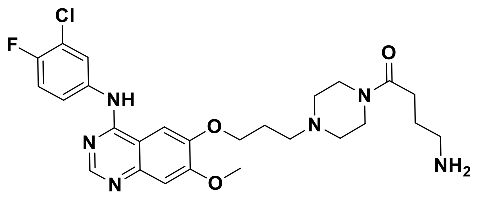

To a stirred solution of 4-azido-1-(4-(3-((4-((3-chloro-4-fluorophenyl)amino)-7-methoxyquinazolin-6-yl)oxy)propyl)piperazin-1-yl)butan-1-one (49.0 mg, 88.0 µmol) in methanol (2mL) was added tin (II) chloride (25.0 mg, 0.132 mmol). The reaction was stirred at room temperature overnight, then concentrated under reduced pressure. The residue was suspended in ethyl acetate (15mL) and washed with 5M aqueous potassium fluoride solution (10 mL). The aqueous layer was further extracted with ethyl acetate (10 mL). The combined organic layers were washed with brine, dried (Na_2SO_4) and concentrated under reduced pressure. The crude material was purified by flash column chromatography using amino silica eluting with methanol (0-20%) in dichloromethane to yield the titled compound as an oil (35mg, 75%). R_f 0.2 (10% MeOH:DCM); m.p. 185-190°C; UV λ_{max} (nm) 335,249,225;^{1}H NMR (500 MHz, Chloroform-d) δ 8.64 (s, 1H), 7.85 (dd, J = 7.8, 3.5 Hz, 1H), 7.57 – 7.53 (m, 1H), 7.27 (s, 1H), 7.23 (s, 1H),7.14 (t, J = 8.7 Hz, 1H), 4.14 (t, J = 6.5 Hz, 2H), 3.97 (s, 3H), 3.60 (t, J = 5.1 Hz, 2H), 3.49 – 3.45 (m, 2H), 2.73 (t, J = 6.9 Hz, 2H), 2.54 (t, J = 7.0 Hz, 2H), 2.44 (t, J = 5.0 Hz, 2H), 2.39 – 2.34 (m, 2H), 2.07 (p, J = 6.7 Hz, 2H), 1.75 (p, J = 7.1 Hz, 2H), 1.25 (m, 2H);^{13}C NMR (126 MHz, Chloroform-d) δ 171.4, 156.3, 155.7, 154.5 (d, J_{C-F} = 184.3 Hz), 153.4, 149.0, 147.5, 135.5, 124.2, 121.79 (d, J_{C-F} = 6.7 Hz), 120.9 (d, J_{C-F} = 18.5 Hz,), 116.6 (d, J_{C-F} = 21.9 Hz), 109.0, 107.9, 101.1, 67.5, 56.2, 54.7, 53.4, 52.8, 45.5, 41.6, 41.6, 30.6, 28.5, 26.4; ^{19}F NMR (471 MHz, Chloroform-d) δ -121.0; HRMS calculated for $C_{26}H_{32}(^{35}Cl)FN_6O_3$ $[M+H]^+$ 531.2281 found 531.3932.

Methyl (*E*)-3-(1H-pyrrol-2-yl)acrylate

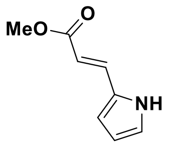

To a stirred solution of pyrrole-2-carboxaldehyde (1.03 g, 10.4 mmol) in dichloromethane (50 mL) was added methyl (triphenylphosphoranylidene)acetate (7.07 g, 20.7 mmol) and the reaction stirred at room temperature for 24 h. The reaction mixture was concentrated under reduced pressure. The crude material was purified by flash column chromatography eluting with ethyl acetate (20-30%) in 40-60 petroleum ether to yield the title compound as an off white solid (60%). ^{1}H NMR (500 MHz, Chloroform-d) δ 8.96 (s, 1H), 7.51 (d, J = 15.9 Hz, 1H), 6.86 (td, J = 1.4, 2.8 Hz, 1H), 6.49 (ddd, J = 1.4, 2.5, 3.8 Hz, 1H), 6.21 (dt, J = 2.5, 3.7 Hz, 1H), 5.98 (d, J = 15.9 Hz, 1H), 3.71 (s, 3H); ^{13}C NMR (126 MHz, Chloroform-*d*) δ 168.3, 134.6, 128.4, 122.6, 114.5, 111.0, 110.7, 51.6.

Data is consistent with what has been reported in the literature.[5]

Methyl 3-(1H-pyrrol-2-yl)propanoate

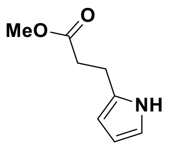

To a stirred solution of methyl (*E*)-3-(1H-pyrrol-2-yl)acrylate (800 mg, 5.29 mmol) in methanol (25 mL) was added 10% palldium on carbon (56.0 mg). The reaction was stirred under hydrogen for 3 h then filtered through Celite and concentrated under redcued pressure. The crude material was purified by flash column chromatography eluting with ethyl acetate (20%) in 40-60 petroleum ether to yield the title compound as pale yellow oil (650 mg, 81%). ^{1}H NMR (500 MHz, Chloroform-d) δ 8.46 (s, 1H), 6.60 (td, J = 1.5, 2.7 Hz, 1H), 6.03 (q,

J = 2.8 Hz, 1H), 5.84 (ddd, J = 1.6, 2.6, 3.3 Hz, 1H), 3.62 (s, 3H), 2.84 (t, J = 6.8 Hz, 2H), 2.57 (t, J = 6.8 Hz, 2H); ^{13}C NMR (126 MHz, Chloroform-d) δ 174.6, 131.0, 116.8, 108.0, 105.5, 51.8, 34.4, 22.6; LRMS (ES$^+$) m/z 154 [M+H]$^+$

Data is consistent with what has been reported in the literature.[5]

4,4-Difluoro-1,3-dimethyl-4-bora-3a,4a-diaza-s-indacene-2-propionic acid methyl ester

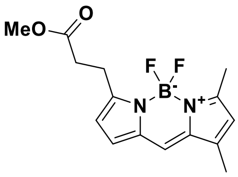

To a stirred solution of methyl 3-(1*H*-pyrrol-2-yl)propanoate (500 mg, 3.26 mmol), 3,5-dimethylpyrrole-2-carboxaldehyde (442 mg 3.59 mmol) in dichloromethane (25 mL) at 0 °C was added phosphorus (v) oxychloride (335 μL, 3.59 mmol) dropwise. After stirring for 30 minutes at 0 °C the reaction was stirred for 7 h at room temperature. The resultant dark solution was cooled down to 0 °C and $BF_3 \cdot OEt_2$ (1.59 mL, 13.0 mmol) and N,N-diisopropyl-N-ethylamine (2.15 mL, 13.0 mmol) were added and the mixture stirred overnight at room temperature. The reaction was quenched with water (50 mL) then filtered through Celite, washing with dichloromethane (50 mL). The filtrate was diluted with dichloromethane (50 mL) and separated. The aqueous layer was extracted with dichloromethane (3 x 30 mL). The combined organic layers were dried (Na_2SO_4) and concentrated under reduced pressure. The crude material was purified by flash column chromatography eluting with dichloromethane to yield the title compound as dark red solid (680 mg, 75%). ^{1}H NMR (500 MHz, Chloroform-d): δ 7.09 (s, 1H), 6.89 (d, J = 3.9 Hz, 1H), 6.27 (d, J = 4.0 Hz, 1H), 6.12 (s, 1H), 3.70 (s, 3H), 3.31 (t, J = 7.6 Hz, 2H), 2.78 (t, J = 7.5 Hz, 2H), 2.58 (s, 3H), 2.26 (s, 3H); ^{13}C NMR (126 MHz, $CDCl_3$): δ 173.2, 160.8, 144.1, 135.5, 133.5, 128.3, 124.1, 120.7, 116.9, 52.0, 33.5, 30.0, 15.2, 11.5; ^{19}F NMR (471 MHz, Chloroform-d) δ -145.1, -145.6; LRMS (ES$^+$) m/z 307 [M+H]$^+$

Data is consistent with what has been reported in the literature.[5]

4,4-difluoro-1,3-dimethyl-4-bora-3a,4a-diaza-s-indacene-2-propionic acid

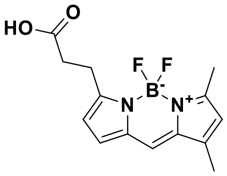

A solution of 4,4-Difluoro-1,3-dimethyl-4-bora-3a,4a-diaza-s-indacene-2-propionic acid methyl ester (650 mg) and concentrated hydrochloric acid in THF and water was stirred at room temperature for 24 h. Dichloromethane (150 mL) was added and the phases separated. The aqueous layer was extracted with dichloromethane (2 × 150 mL). The combined organic layers were washed with brine, dried ($MgSO_4$) and concentrated under reduced pressure. The crude material was purified by flash column chromatography eluting with dichloromethane (1% acetic acid) to yield the title compound as red solid (486 mg, 78%). ^{1}H NMR (500 MHz, DMSO-d^6): δ = 12.30 (s, 1H), 7.70 (s, 1H), 7.09 (d, J = 4.0 Hz, 1H), 6.38 (d, J = 4.0 Hz, 1H), 6.31 (s, 1H), 3.10–3.05 (t, J = 7.1 Hz, 2H), 2.64 (t, J = 8.5 Hz, 2H), 2.47 (s, 3H), 2.26 ppm (s, 3H); ^{13}C NMR (126 MHz, DMSO-d^6): δ=173.4, 159.5, 156.9, 144.3, 134.5, 133.0, 128.8, 125.4, 120.4, 116.5, 32.3, 23.5, 14.5, 11.0; LRMS (ES$^+$) m/z 293 [M+H]$^+$

Data is consistent with what has been reported in the literature.[5]

N-(4-(4-(3-((4-((3-chloro-4-fluorophenyl)amino)-7-methoxyquinazolin-6-yl)oxy)propyl)piperazin-1-yl)-4-oxobutyl)-3-(5,5-difluoro-7,9-dimethyl-5H-5l4,6l4-dipyrrolo[1,2-c:2',1'-f][1,3,2]diazaborinin-3-yl)propenamide, 6

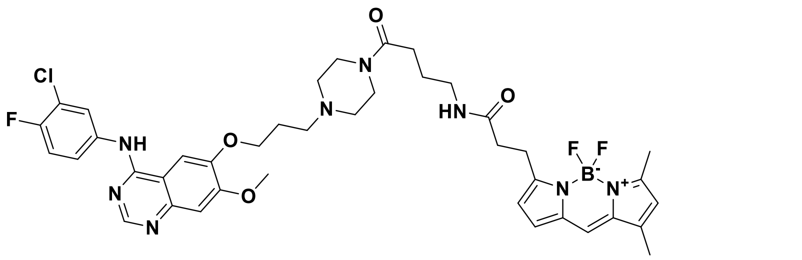

To a stirred solution of 4,4-difluoro-1,3-dimethyl-4-bora-3a,4a-diaza-s-indacene-2-propionic acid (38.0 mg, 0.136 mmol) in DMF (2mL) was added N,N-diisopropyl-N-ethylamine (50.1 mg, 0.389 mmol) and HATU (74.0 mg, 0.194 mmol). The reaction was stirred at room temperature for 30 minutes then 4-amino-1-(4-(3-((4-((3-chloro-4-fluorophenyl)amino)-7-methoxyquinazolin-6-yl)oxy)propyl)piperazin-1-yl)butan-1-one (69.0 mg, 0.129 mmol) was added and the reaction mixture stirred overnight. The reaction mixture was concentrated under reduced pressure. The residue was dissolved in dichloromethane (30 mL), washed with saturated aqueous sodium hydrogen carbonate solution (2 x 10 mL). The aqueous layer was back extracted, the combined organic layers were dried (Na_2SO_4) and concentrated under reduced pressure. The crude material was purified by flash column chromatography using by amino silica, eluting with methanol in ethyl acetate to yield the title compound as red solid (62%). m.p 235 °C; UV λ_{max} (nm) 510,341; IR: ν_{max}/cm^{-1} 3282 (m, N-H) ,1600 (s, C=O), 1497 (s, B-F); ^{1}H NMR (500 MHz, Chloroform-d) δ 8.63 (s, 1H), 8.00 (s, 1H), 7.89 (dd, J = 6.6, 2.7 Hz, 1H), 7.57 (dt, J = 9.1, 3.5 Hz, 1H), 7.37 (s, 1H), 7.22 (s, 1H), 7.10 (t, J = 8.8 Hz, 1H), 7.03 (s, 1H), 6.82 (d, J = 4.0 Hz, 1H), 6.22 (d, J = 4.0 Hz, 1H), 6.14 (t, J = 5.9 Hz, 1H), 6.03 (s, 1H), 4.19 (t, J = 6.6 Hz, 2H), 3.98 (s, 3H), 3.70 – 3.65 (m, 2H), 3.48 (s, 2H), 3.25 (p, J = 7.9, 7.1 Hz, 4H), 2.66 (t, J = 6.8 Hz, 2H), 2.62 – 2.57 (m, 2H), 2.56 – 2.50 (m, 3H), 2.49 (s, 3H), 2.30 (t, J = 6.8 Hz, 2H), 2.20 (s, 3H), 2.11 (t, J = 6.7 Hz, 2H), 1.80 (p, J = 6.7 Hz, 2H); ^{13}C NMR (126 MHz, Chloroform-d) δ 172.0, 171.0, 160.4, 157.0, 156.3, 155.5 (d, $J_{C\text{-}F}$ = 247 Hz), 155.1, 153.4, 148.8, 147.5, 144.1, 135.6, 135.2, 133.3, 128.1, 124.0, 123.8, 121.67-121.62 (d, $J_{C\text{-}F}$ = 7.1 Hz), 120.9, 120.72, 120.5, 116.9, 116.4 ($J_{C\text{-}F}$ = 21 Hz), 109.1, 107.8, 101.8, 67.5, 56.2, 54.6, 53.0, 52.6, 45.1, 41.2, 39.2, 35.7, 30.3, 25.9, 24.8,14.9, 11.3; ^{11}B NMR (160 MHz, Chloroform-d) δ 0.91 (t, *J* = 34.4 Hz).; ^{19}F NMR (471 MHz, Chloroform-d) δ -121.4, -144.3. HRMS calculated for $C_{40}H_{45}B(^{35}Cl)F_3N_8O_4$ [M+H]+ 805.3370 found 805.3367.

1-(4-((4-((3-Chloro-2-fluorophenyl)amino)quinazolin-6-yl)oxy)piperidin-1- yl)prop-2-en-1-one, 7

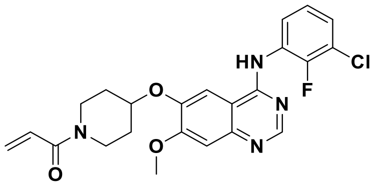

To a solution of N-(5-chloro-2-fluorophenyl)-6-(piperidin-4-yloxy)quinazolin-4-amine synthesised according to reference[6] (50 mg, 0.13 mmol) in THF (1.0 mL) was added acryloyl chloride (17 mg, 0.19 mmol) and $NaHCO_3$ (19 mg, 0.23 mmol) under an atmosphere of N_2 and allowed to stir at 0 °C for 30 min then left to stir at room temperature overnight. After this time the reaction mixture was concentrated in vacuo and purified via flash column chromatography (eluent: 0-10% MeOH in DCM) to give 1-(4-((4-((3-chloro-2-fluorophenyl)amino)quinazolin-6-yl)oxy)piperidin-1-yl)prop-2-en-1-one (41 mg, 0.09 mmol, 71%) as a white solid. R_f = 0.15 (5% MeOH in DCM); mp 213-215 °C; UV λ_{max} (nm) 332, 289; IR: ν_{max}/cm^{-1} 2921 (s, C-H alkene), 2852 (s, C-H alkane), 1690 (m, C=O amide), 1573 (m, C=C aromatic); ^{1}H NMR (500 MHz, $CDCl_3$): δ_H 8.75 (s, 1H), 8.51 dd, $J_{H\text{-}H}$ = 7.0, $J_{H\text{-}F}$ = 3.4 Hz, 1H), 7.93 (d, *J* = 9.1 Hz, 1H), 7.52 (dd, *J* = 9.10, 2.6 Hz, 1H), 7.25 (d, *J* = 2.6 Hz, 1H), 7.19-7.15 (m, 2H), 6.62 (dd, *J* = 16.8, 10.6 Hz, 1H), 6.31 (dd, *J* = 16.8, 1.9 Hz, 1H), 5.72 (dd, *J* = 10.6, 1.9 Hz, 1H), 4.80-4.73 (m, 1H), 3.83-3.63 (m, 4H), 2.04-1.96 (m, 4H); ^{13}C NMR (126 MHz, $CDCl_3$): δ_C 165.6, 156.2, 155.6, 152.8, 145.8, 131.1, 128.0, 127.5, 125.4 (d, $J_{C\text{-}F}$ = 6.5 Hz), 125.0 (d, $J_{C\text{-}F}$ = 4.8 Hz), 124.9 (d, $J_{C\text{-}F}$ = 6.6 Hz), 124.6, 121.5, 121.0, 120.9, 116.0, 103.3, 72.5, 31.9, 31.1; ^{19}F NMR (470 MHz, $CDCl_3$): δ_F -131.83; LRMS (ES$^+$) m/z 427.3 $[M(^{35}Cl)+H]^+$ and 429.3 $[M(^{37}Cl)+H]^+$.

1-(4-((4-((3-Chloro-4-fluorophenyl)amino)-7-methoxyquinazolin-6-yl)oxy)piperidin-1-yl)prop-2-en-1-one, 8

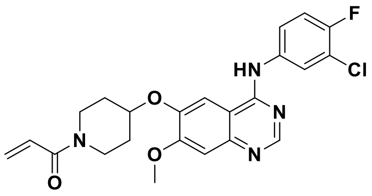

To a solution of N-(3-chloro-4-fluorophenyl)-7-methoxy-6-(piperidin-4-yloxy)quinazolin-4-amine hydrochloride synthesised according to reference[6] (100 mg, 0.23 mmol) in THF (1.0 mL) was added acryloyl chloride (29 mg, 0.32 mmol) and $NaHCO_3$ (32 mg, 0.39 mmol) under an atmosphere of N_2 and allowed to stir at 0 °C for 30 min then left to stir at room temperature overnight. After this time, the reaction mixture was concentrated in vacuo and purification performed via flash column chromatography (eluent: 0-10% MeOH in DCM) to give 1-(4-((4-((3-chloro-4-fluorophenyl)amino)-7-methoxyquinazolin-6- yl)oxy)piperidin-1-yl)-prop-2-en-1-one (9.0 mg, 20 µmol, 9%) as a white solid. R_f = 0.18 (5% MeOH in DCM); mp 217-219 °C; UV λ_{max} (nm) 340, 332, 248; IR: ν_{max}/cm^{-1} 2920 (s, C-H alkene), 2851 (s, C-H alkane), 1737 (w, C=O amide), 1598 (m, C=C alkene), 1575 (m, C=C aromatic); ^{1}H NMR (500 MHz, $CDCl_3$): δ_H 8.59 (s, 1H), 7.87 (dd, J_{H-F} = 6.56 Hz, J_{H-H} = 2.66 Hz, 1H), 7.60 (ddd, J_{H-H} = 8.89, 2.70 Hz, J_{H-F} = 4.10 Hz, 1H), 7.50 (s, 1H), 7.25 (s, 1H), 7.21 (s, 1H), 7.14 (d, J = 8.86 Hz, 1H), 6.61 (dd, J = 16.83, 10.61 Hz, 1H), 6.27 (dd, J = 16.83, 1.75 Hz, 1H), 5.70 (dd, J = 10.61, 1.75 Hz, 1H), 4.70-4.63 (m, 1H), 3.95 (s, 3H), 3.93-3.52 (m, 4H), 2.05-1.82 (m, 4H); ^{13}C NMR (126 MHz, $CDCl^3$): δ^C 165.8, 156.7, 156.6, 153.8, 153.4, 147.2, 146.6, 176 135.47 (d, J_{C-F} = 6.75 Hz), 128.3, 127.5, 124.3, 124.2, 121.9 (d, J_{C-F} = 3.20 Hz), 116.6, 116.4, 107.7, 107.6, 74.0, 56.2, 42.7, 39.0; ^{19}F NMR (470 MHz, $CDCl_3$): δ_F -125.10; LRMS (ES$^+$) m/z 457.3 [M(^{35}Cl)+H]+ and 459.3 [M(^{37}Cl)+H]$^+$.

1-(4-((4-((5-Chloro-2-fluorophenyl)amino)-7-methoxyquinazolin-6-yl)oxy)piperidin-1-yl)prop-2-en-1-one, 9

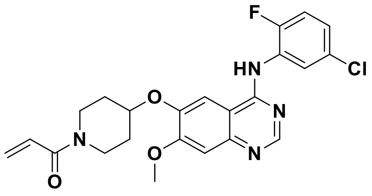

To a solution of N-(5-chloro-2-fluorophenyl)-7-methoxy-6-(piperidin-4-yloxy)quinazolin-4-amine synthesised according to reference[6] (50 mg, 0.11 mmol) in THF (1.0 mL) was added acryloyl chloride (14 mg, 0.16 mmol) and $NaHCO_3$ (16 mg, 0.19 mmol) under an atmosphere of N_2 and allowed to stir at 0 °C for 30 min then left to stir at room temperature overnight. After this time the reaction mixture was concentrated in vacuo and purification performed via flash column chromatography (eluent: 0-10% MeOH in DCM) to give 1-(4-((4-((5-chloro-2-fluorophenyl)amino)-7-methoxyquinazolin-6-yl)oxy)piperidin-1-yl)prop-2-en-1-one (32 mg, 70 µmol, 62%) as a white solid. R_f = 0.12 (5% MeOH in DCM); mp 211-213 °C; UV λ_{max} (nm) 330, 249, 228; IR: ν_{max}/cm^{-1} 2922 (s, C-H alkene), 2852 (m, C-H alkane), 1690 (m, C=O amide), 1636 (m, C=C alkene), 1575 (m, C=C aromatic); ^{1}H NMR (500 MHz, CDCl3): δH 8.75 (s, 1H), 8.72 (dd, J = 7.1, 2.55 Hz, 1H), 7.37 (s, 1H), 7.32 (s, 1H), 7.22 (s, 1H), 7.11 (dd, J_{H-F} = 10.62 Hz, J_{H-H} = 8.80 Hz, 1H), 7.04 (ddd, J_{H-H} = 8.80, 2.55 Hz, J_{H-F} = 4.50 Hz), 6.61 (dd, J = 16.80, 10.61 Hz, 1H), 6.30 (dd, J = 16.80, 1.90 Hz, 1H), 5.71 (dd, J = 10.62, 1.90 Hz, 1H), 4.74-4.67 (m, 1H), 4.01 (s, 3H), 3.90-3.56 (m, 4H), 2.01-1.96 (m, 4H); ^{13}C NMR (126 MHz, $CDCl_3$): δ_C 165.6, 156.8, 155.5, 153.7, 148.3, 147.2, 129.9 (d, J_{C-F} = 9.04 Hz), 127.9, 128.3, 127.6, 123.4, 122.6 (d, J_{C-F} = 6.55 Hz,), 115.8 (d, J_{C-F} = 37.89 Hz), 115.7, 109.2, 108.8, 105.8, 74.6, 56.3, 44.0, 42.7; ^{19}F NMR (470 177 MHz, $CDCl_3$): δ_F -133.5; LRMS (ES$^+$) m/z 457.3 [M(^{35}Cl)+H]$^+$ and 459.3 [M(^{37}Cl)+H]$^+$.

1-(4-((4-((5-Chloro-2-fluorophenyl)amino)-7-methoxyquinazolin-6- yl)oxy)piperidin-1-yl)prop-2-yn-1-one, 10

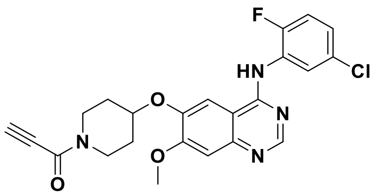

To a solution of HATU (227 mg, 0.60 mmol), N-(5-chloro-2-fluorophenyl)-7-methoxy6-(piperidin-4-yloxy)quinazolin-4-amine synthesised according to reference[6] (200 mg, 0.50 mmol) and propiolic acid (38 mg, 0.55 mmol) in DMA (2.5 mL) was added DIPEA (257 mg, 1.99 mmol) under an atmosphere of N_2 and allowed to stir at room temperature for 2 h before being poured over water (ca. 30 mL). The resulting precipitate was filtered and dried in vacuo to give a yellow solid. Purification was performed via flash column chromatography (eluent: 0-10% MeOH in DCM) to give 1-(4-((4-((5-chloro-2-fluorophenyl)amino)-7-methoxyquinazolin-6-yl)oxy)piperidin-1-yl)prop-2-yn-1-one (106 mg, 0.23 mmol, 47%) as a yellow solid. R_f = 0.12 (5% MeOH in DCM); mp 201-203 °C; UV λ_{max} (nm) 331, 248, 226; IR: ν_{max}/cm^{-1} 2931 (s, C-H aromatic), 2856 (m, C-H alkane), 2101 (w, C≡C alkyne), 1616 (s, C=O amide), 1576 (m, C=C aromatic); ^{1}H NMR (500 MHz, DMSO-d$_6$): δ_H 9.50 (s, 1H), 8.40 (s, 1H), 7.89 (s, 1H,), 7.71 (dd, J_{H-F} = 6.64 Hz, J_{H-H} 2.50 Hz, 1H), 7.44-7.32 (m, 2H), 7.24 (s, 1H), 4.85-4.74 (m, 1H), 4.56 (s, 1H), 3.95 (s, 3H), 3.83-3.43 (m, 4H), 2.18-1.63 (m, 4H); ^{13}C NMR (126 MHz, DMSO-d$_6$): δ_C 156.8, 155.6, 153.6, 151.8, 148.1, 147.0, 129.9, 128.2, 123.7, 123.1, 115.9 (d, J_{C-F} = 21.20 Hz), 109.2, 108.6, 106.1, 79.4, 75.4, 74.1, 56.3, 43.6, 37.9; ^{19}F NMR (470 MHz, DMSO-d6): δ_F - 121.10; LRMS (ES+) m/z 455.0 [M(^{35}Cl)+H]$^+$ and 457.0 [M(^{37}Cl)+H]$^+$.

1-(4-((4-((3-Chloro-4-fluorophenyl)amino)-7-methoxyquinazolin-6-yl)oxy)piperidin-1-yl)prop-2-yn-1-one, 11

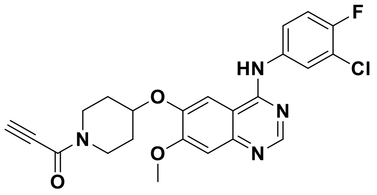

To a solution of HATU (312 mg, 0.82 mmol), N-(3-chloro-4-fluorophenyl)-7-methoxy-6-(piperidin-4-yloxy)quinazolin-4-amine hydrochloride synthesised according to reference[6] (300 mg, 0.68 mmol) and propiolic acid (52 mg, 0.75 mmol) in DMA (3.4 mL) was added DIPEA (353 mg, 2.73 mmol) under an atmosphere of N_2 and allowed to stir at room temperature for 2 h before being poured over water (ca. 30 mL). The resulting precipitate was filtered and dried in vacuo to give 1-(4-((4-((3-chloro-4-fluorophenyl)amino)-7-methoxyquinazolin-6-yl)oxy)piperidin-1-yl)prop-2-yn-1-one (233 mg, 0.51 mmol, 75%) as a yellow solid. R_f = 0.14 (5% MeOH in DCM); mp 206-208 °C; UV λ_{max} (nm) 332, 247; IR: ν_{max}/cm^{-1} 2915 (s, C-H aromatic), 2847 (m, C-H alkane), 2105 (w, C≡C alkyne), 1613 (s, C=O amide), 1585 (m, C=C aromatic); ^{1}H NMR (500 MHz, DMSO-d$_6$): δ_H 9.52 (s, 1H), 8.52 (s, 1H), 8.12 (dd, J_{H-F} = 6.85, J_{H-H} = 2.64 Hz, 1H), 7.96 (s, 1H), 7.79 (ddd, J_{H-H} = 8.95, 2.68 Hz, J_{H-F} = 4.28 Hz, 1H), 7.46 (d, J = 9.00 Hz, 1H), 7.26 (s, 1H), 4.85-4.78 (m, 1H), 4.56 (s, 1H), 3.96 (s, 3H), 3.76-3.48 (m, 4H), 2.13-1.77 (m, 4H); ^{13}C NMR (126 MHz, $CDCl_3$): δ_C 157.0, 156.5, 153.3, 152.5, 146.9, 136.4, 125.0, 123.70 (d, J_{C-F} = 7.56 Hz), 121.2, 119.5, 119.3, 117.0 (d, J_{C-F} = 21.42 Hz), 108.7, 106.8, 106.2, 82.7, 76.1, 73.8, 56.6, 38.8, 30.9; ^{19}F NMR (470 MHz, DMSO-d$_6$): δ_F -123.05; LRMS (ES$^+$) *m/z* 455.0 [M(^{35}Cl)+H]$^+$ and 457.0.

1-(4-((4-((3-Chloro-2-fluorophenyl)amino)-7-methoxyquinazolin-6-yl)oxy)piperidin-1-yl)prop-2-yn-1-one, 12

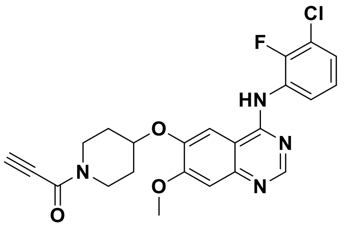

To a solution of HATU (227 mg, 0.60 mmol), N-(3-chloro-2-fluorophenyl)-7-methoxy6-piperidin-4-yloxy)quinazolin-4-amine synthesised according to reference[6] (200 mg, 0.50 mmol) and propiolic acid (38 mg, 0.55 mmol) in DMA (2.4 mL) was added DIPEA (257 mg, 1.99 mmol) under an atmosphere of N_2 and allowed to stir at room temperature for 2 h before being poured over water (ca. 30 mL). The resulting precipitate was filtered and dried in vacuo to give a yellow solid. Purification was performed via flash column chromatography (eluent: 0- 10% MeOH in DCM) to give 1-(4-((4-((3-chloro-2-fluorophenyl)amino)-7-methoxyquinazolin-6-yl)oxy)piperidin-1-yl)prop-2-yn-1-one (84 mg, 0.18 mmol, 37%) as a light yellow solid. R_f = 0.13 (5% MeOH in DCM); mp 202-204 °C; UV λ_{max} (nm) 339, 281, 210; IR: ν_{max}/cm^{-1} 2926 (s, C-H aromatic), 2851 (m, C-H alkane), 2100 (w, C≡C alkyne), 1614 (s, C=O amide), 1575 (m, C=C aromatic); ^{1}H NMR (500 MHz, DMSO-d$_6$): δ_H 9.59 (s, 1H), 8.39 (s, 1H), 7.90 (s, 1H), 7.52 (m, 2H), 7.30 (dd, J_{H-H} = 8.17 Hz, J_{H-F} = 1.33 Hz, 1H), 7.25 (s, 1H), 4.82-4.76 (m, 1H), 4.56 (s, 1H), 3.96 (s, 3H), 3.85-3.45 (m, 4H), 2.14-1.64 (m, 4H); ^{13}C NMR (126 MHz, CDCl$_3$): δ_C 156.8, 153.7, 151.8, 150.8, 147.2, 131.8, 126.9, 125.8, 124.5 (d, J_{C-F} = 5.04 Hz), 122.0, 116.4, 114.7, 114.3, 108.6, 106.1, 79.5, 75.4, 74.0, 56.3, 43.6, 37.9; ^{19}F NMR (470 MHz, DMSO-d$_6$): δ_F -120.47; LRMS (ES$^+$) m/z 455.0 [M(^{35}Cl)+H]$^+$ and 457.0 [M(^{37}Cl)+H]$^+$.

tert-Butyl 4-((4-((3,4-dichloro-2-fluorophenyl)amino)-7-methoxyquinazolin-6-yl) oxy)piperidine-1-carboxylate

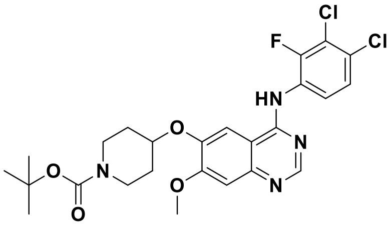

To a round bottom flask charged with tert-butyl 4-((4-chloro-7-methoxyquinazolin-6-yl)oxy)piperidine-1-carboxylate (116 mg, 0.29 mmol) and 3,4-dichloro-2-fluoroaniline (53 mg, 0.29 mmol) was added DMF (1.5 mL) and stirred at 75 °C for 1.5 h before concentrating in vacuo to give a yellow solid. Purification was performed via flash column chromatography (eluent: 0-100% EtOAc in n-heptane followed by 0-50% MeOH in EtOAc) to give tert-butyl 4-((4-((3,4-dichloro-2-fluorophenyl)amino)-7-methoxyquinazolin-6-yl)oxy)piperidine-1-carboxylate (69 mg, 0.13 mmol, 44%) as an off-white solid. R_f = 0.12 (10% MeOH in DCM); ^{1}H NMR (500 MHz, CDCl$_3$): δ_H 8.71 (s, 1H), 8.52 (t, J = 9.00 Hz, 1H), 7.34 (dd, J_{H-H} = 9.00, J_{H-F} 6.61 Hz, 1H), 7.31 (s, 1H), 7.20 (s, 1H), 7.17 (s, 1H), 4.69-4.54 (m, 1H), 4.02 (s, 3H), 3.89-3.74 (m, 2H), 3.35 (m, 2H), 2.03-1.96 (m, 2H), 1.91-1.83 (m, 2H), 1.48 (s, 9H); ^{13}C NMR (126 MHz, CDCl$_3$): δ_C 156.9, 155.4, 154.8, 153.5, 148.2, 147.5, 127.4, 127.0, 125.2, 125.2 (d, J_{C-F} = 22.52 Hz), 121.0, 109.2, 108.7, 107.3, 105.1, 80.0, 76.0, 56.3, 32.1, 30.1, 28.5; ^{19}F NMR (470 MHz, DMSO-d$_6$) : δ_F -113.61; LRMS (ES+) m/z 537.0 [M(^{35}Cl)(^{35}Cl)+H]$^+$, 539.1 [M(^{35}Cl)(^{37}Cl)+H]$^+$, [M(^{37}Cl)(^{35}Cl)+H]+ and 541.1 [M(^{37}Cl)(^{37}Cl)+H]+.

Data is in accordance with that reported in the literature.[7]

(E)-3-(3-(4-((4-((3,4-Dichloro-2-fluorophenyl)amino)-7-methoxyquinazolin-6-1-(4-((4-((3,4-Dichloro-2-fluorophenyl)amino)-7-methoxyquinazolin-6- yl)oxy)piperidin-1-yl)prop-2-yn-1-one, 13

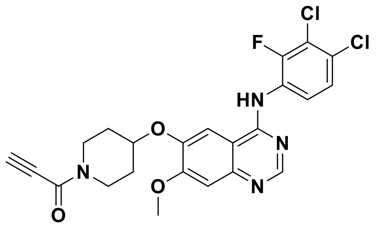

To a stirred solution of tert-butyl 4-((4-((3,4-dichloro-2-fluorophenyl)amino)-7-methoxyquinazolin-6-yl)oxy)piperidine-1-carboxylate (60 mg, 0.14 mmol) in DCM (0.5 mL) was added 2,2,2-trifluoroacetic acid (63.7 mg, 0.56 mmol) in one portion and allowed to stir at room temperature for 1 h before passing through a SCX column and washing with 7 M NH_3 in MeOH. The resulting filtrate was concentrated in vacuo to give a white solid. To this was added DMA (0.7 mL), followed by DIPEA (70.9 mg, 0.55 mmol), HATU (63 mg, 0.16 mmol), and propiolic acid (10.57 mg, 0.15 mmol) under an atmosphere of N_2 and allowed to stir at room temperature for 2 h before being poured over water (ca. 30 mL). The resulting precipitate was filtered and dried in vacuo to give 1-(4-((4-((3,4-dichloro-2-fluorophenyl)amino)-7-methoxyquinazolin-6-yl)oxy)piperidin1-yl)prop-2-yn-1-one (61 mg, 0.13 mmol, 91%) as a red solid. R_f = 0.12 (10% MeOH in DCM); mp 203-205 °C; UV λ_{max} (nm) 330, 248; IR: ν_{max}/cm^{-1} 2921 (s, C-H aromatic), 2852 (m, C-H alkane), 2101 (w, C≡C alkyne), 1614 (s, C=O amide), 1586 (m, C=C aromatic); ^{1}H NMR (500 MHz, $CDCl_3$): δ_H 8.71 (s, 1H), 8.50 (dd, J_{H-H} = 9.00 Hz, J_{H-F} = 7.82 Hz, 1H), 7.35 (d, J_{H-H} = 9.06 Hz, J_{H-F} 2.0 Hz, 1H), 7.33 (s, 1H), 7.23 (s, 1H), 7.20 (s, 1H), 4.78-4.71 (m, 1H), 4.02 (s, 3H), 3.92-3.78 (m, 2H), 3.14 (s, 1H), 3.06-2.98 (m, 4H) 2.07-1.95 (m, 2H); ^{13}C NMR (126 MHz, DMSO-d$_6$): δ_C 159.5, 158.5, 153.6, 152.0, 148.5, 147.3 (d, J_{C-F} = 17.65 Hz), 127.9, 127.2, 125.3, 122.9, 121.6, 109.2, 108.9, 107.7, 105.9, 79.5, 75.5, 74.2, 56.3, 43.6, 37.8; ^{19}F NMR (470 MHz, $CDCl_3$): δ_F -126.03; LRMS (ES$^+$) m/z 488.0 [M(^{35}Cl)(^{35}Cl)+H]$^+$, 490.1 [M(^{35}Cl)(^{37}Cl)+H]$^+$, [M(^{37}Cl)(^{35}Cl)+H]$^+$ and 492.1

1-(4-((4-((3-Chloro-2-fluorophenyl)amino)quinazolin-6-yl)oxy)piperidin-1-yl)prop-2-en-1-one, 14

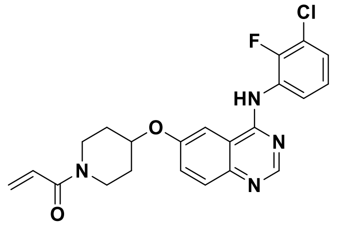

To a solution of N-(5-chloro-2-fluorophenyl)-6-(piperidin-4-yloxy)quinazolin-4-amine (50 mg, 0.13 mmol) in THF (1.0 mL) was added acryloyl chloride (17 mg, 0.19 mmol) and $NaHCO_3$ (19 mg, 0.23 mmol) under an atmosphere of N_2 and allowed to stir at 0 °C for 30 min then left to stir at room temperature overnight. After this time the reaction mixture was concentrated in vacuo and purified via flash column chromatography (eluent: 0-10% MeOH in DCM) to give 1-(4-((4-((3-chloro-2-fluorophenyl)amino)quinazolin-6-yl)oxy)piperidin-1-yl)prop-2-en-1-one (41 mg, 0.09 mmol, 71%) as a white solid. R_f = 0.15 (5% MeOH in DCM); mp 213-215 °C; UV λ_{max} (nm) 332, 289; IR: ν_{max}/cm^{-1} 2921 (s, C-H alkene), 2852 (s, C-H alkane), 1690 (m, C=O amide), 1573 (m, C=C aromatic); ^{1}H NMR (500 MHz, $CDCl_3$): δ_H 8.75 (s, 1H), 8.51 dd, J_{H-H} = 7.00 Hz, J_{H-F} = 3.42 Hz, 1H), 7.93 (d, J = 9.10 Hz, 1H), 7.52 (dd, J = 9.10, 2.57 Hz, 1H), 7.25 (d, J = 2.57 Hz, 1H), 7.19-7.15 (m, 2H), 6.62 (dd, J = 16.82, 10.60 Hz, 1H), 6.31 (dd, J = 16.82, 1.90 Hz, 1H), 5.72 (dd, J = 10.60, 1.90 Hz, 1H), 4.80-4.73 (m, 1H), 3.83-3.63 (m, 4H), 2.04-1.96 (m, 4H); ^{13}C NMR (126 MHz, $CDCl_3$): δ_C 165.6, 156.2, 155.6, 152.8, 145.8, 131.1, 128.0, 127.5, 125.4 (d, J_{C-F} = 6.54 Hz), 125.0 (d, J_{C-F} = 4.85 Hz), 124.9 (d, J_{C-F} = 6.62 Hz), 124.6, 121.5, 121.0, 120.9, 116.0, 103.3, 72.5, 31.9, 31.1; ^{19}F NMR (470 MHz, $CDCl_3$): δ_F -131.83; LRMS (ES$^+$) m/z 427.3 [M(^{35}Cl)+H]$^+$ and 429.3 [M(^{37}Cl)+H]$^+$.

1-(4-((4-((3-Chloro-2-fluorophenyl)amino)quinazolin-6-yl)oxy)piperidin-1-yl)prop-2-yn-1-one, 15

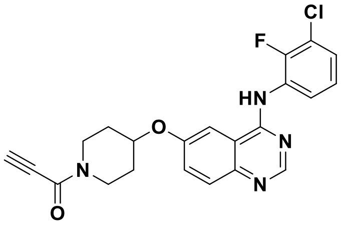

To a solution of HATU (245 mg, 0.64 mmol), N-(3-chloro-2-fluorophenyl)-6-(piperidin4-yloxy)quinazolin-4-amine (200 mg, 0.54 mmol) and propiolic acid (41 mg, 0.59 mmol) in DMA (2.7 mL) was added DIPEA (277 mg, 2.15 mmol) under an atmosphere of N_2 and allowed to stir at room temperature for 2 h before being poured over water (ca. 30 mL). The resulting precipitate was filtered and dried in vacuo to give a yellow solid. Purification was performed via flash column chromatography (0-10% MeOH in DCM) to give 1-(4-((4-((3-chloro-2-fluorophenyl)amino)quinazolin-6-yl)oxy)piperidin-1-yl)prop-2-yn-1-one (118 mg, 0.28 mmol, 52%) as a light yellow solid. R_f = 0.15 (5% MeOH in DCM); mp 207-209 °C; UV λ_{max} (nm) 339, 281, 210; IR: ν_{max}/cm^{-1} 2921 (s, CH aromatic), 2851 (m, C-H alkane), 2099 (w, C≡C alkyne), 1606 (s, C=O amide), 1553 (m, C=C aromatic); ^{1}H NMR (500 MHz, DMSO-d$_6$): δ_H 9.76 (s, 1H), 8.43 (s, 1H), 7.93 (d, *J* = 2.52 Hz, 1H), 7.78 (d, *J* = 9.11 Hz, 1H), 7.59 (dd, *J* = 9.11, 2.52 Hz, 1H), 7.53 (m, 2H), 7.31 (ddd, $J_{H\text{-}H}$ = 8.23, 7.40 Hz, $J_{H\text{-}F}$ = 1.08 Hz, 1H), 4.89-4.83 (m, 1H), 4.57 (s, 1H), 4.06-3.44 (m, 4H), 2.16-1.63 (m, 4H); ^{13}C NMR (126 MHz, DMSO-d$_6$): δ_C 157.9, 155.9, 152.0, 150.9, 148.9, 130.9, 128.3, 125.2, 125.0, 124.6 (d, $J_{C\text{-}F}$ = 4.90 Hz), 122.0, 121.0 (d, $J_{C\text{-}F}$ = 16.40 Hz), 106.9, 103.5, 103.4, 79.5, 75.3, 72.3, 43.5, 37.8; ^{19}F NMR (470 MHz, DMSO-d$_6$): δ_F -120.23; LRMS (ES$^+$) m/z 425.0 [M(^{35}Cl)+H]$^+$ and 427.0 [M(^{37}Cl)+H]$^+$.

tert-Butyl (R)-3-((4-((3-chloro-4-fluorophenyl)amino)-7-methoxyquinazolin-6-yl)oxy)pyrrolidine-1-carboxylate

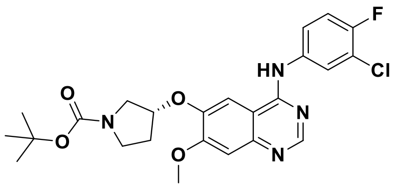

To a stirred solution of 4-((3-chloro-4-fluorophenyl)amino)-7-methoxyquinazolin-6-ol (200 mg, 0.63 mmol) in DMF (1.63 mL) was added potassium carbonate (135 mg, 0.98 mmol) and allowed to stir at room temperature for 1.5 h before addition of tert-butyl (S)-3-((methylsulfonyl)oxy)pyrrolidine-1-carboxylate (130 mg, 0.49 mmol) and heated overnight at 80 °C. After this time, the reaction mixture was concentrated in vacuo and purification performed via flash column chromatography (eluent: 0-10% MeOH in DCM) to give tert-butyl (R)-3-((4-((3-chloro-4-fluorophenyl)amino)-7-methoxyquinazolin-6-yl)oxy)pyrrolidine-1-carboxylate (142 mg, 0.29 mmol, 59%) as an off-white solid. R_f = 0.10 (10% MeOH in DCM); mp 202-204 ºC; $[\alpha]_D^{25.6}$ = +16.7º (c 0.16, EtOH); UV λ_{max} (nm) 331, 248; IR: ν_{max}/cm^{-1} 3323 (m, N-H), 2975 (m, C-H aromatic), 1659 (s, C=O carbamate), 1498 (s, C=C aromatic); ^{1}H NMR (500 MHz, $CDCl_3$): δ_H 8.67 (s, 1H), 7.91 (s, 1H), 7.60 (dd, $J_{H\text{-}F}$ = 6.56 Hz, $J_{H\text{-}H}$ = 2.66 Hz, 1H), 7.52 (ddd, $J_{H\text{-}H}$ = 8.89, 2.70 Hz, $J_{H\text{-}F}$ = 4.10 Hz, 1H), 7.21 (s, 1H), 7.18 (s, 1H), 7.14 (dd, *J* = 8.86, 2.66 Hz, 1H), 5.11 (m, 1H), 4.00 (s, 3H), 3.73-3.41 (m, 4H), 2.27 (m, 2H), 1.48 (s, 9H); ^{13}C NMR (126 MHz, CD_3OD): δ_C 156.7, 156.0, 155.6, 154.8, 148.0, 146.6, 124.3, 122.1 (d, $J_{C\text{-}F}$ = 7.56 Hz), 121.9, 120.8, 120.6, 116.4 (d, $J_{C\text{-}F}$ = 21.42 Hz), 109.0, 108.8, 107.0, 106.4, 80.1, 77.5, 56.0, 50.7, 44.2, 43.8, 28.5; ^{19}F NMR (470 MHz, DMSO-d$_6$): δ_F -121.20; LRMS (ES$^+$) m/z 487.3 [M(^{35}Cl)+H]$^+$ and 489.1 [M(^{37}Cl)+H]$^+$.

tert-Butyl (S)-3-((4-((3-chloro-4-fluorophenyl)amino)-7-methoxyquinazolin-6-yl)oxy)pyrrolidine-1-carboxylate

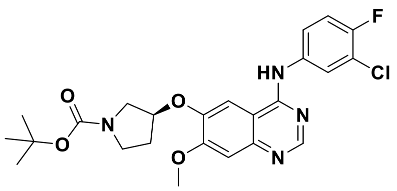

To a stirred solution of 4-((3-chloro-4-fluorophenyl)amino)-7-methoxyquinazolin-6-ol (200 mg, 0.63 mmol) in DMF (1.63 mL) was added potassium carbonate (135 mg, 0.98 mmol) and allowed to stir at room temperature for 1.5 h before addition of tert-butyl (R)-3-((methylsulfonyl)oxy)pyrrolidine-1-carboxylate (130 mg, 0.49 mmol) and heated overnight at 80 °C. After this time, the reaction mixture was concentrated in vacuo and purification performed via flash column chromatography (eluent: 0-10% MeOH in DCM) to give tert-butyl (S)-3-((4-((3-chloro-4-fluorophenyl)amino)-7-methoxyquinazolin-6-yl)oxy)pyrrolidine-1-carboxylate (141 mg, 0.29 mmol, 65%) as an off-white solid. R_f = 0.10 (10% MeOH in DCM); mp 201-203 ºC; $[\alpha]_D^{25.6}$ = -7.0º (c 0.20, EtOH); UV λ_{max} (nm) 331, 248; IR: ν_{max}/cm^{-1} 3318 (m, N-H), 2974 (m, C-H aromatic), 1660 (s, C=O carbamate), 1497 (s, C=C aromatic); ^{1}H NMR (500 MHz, CDCl3): δH 8.67 (s, 1H), 7.91 (s, 1H), 7.60 (dd, J_{H-F} = 6.56 Hz, J_{H-H} = 2.66 Hz, 1H), 7.52 (ddd, J_{H-H} = 8.89, 2.70 Hz, J_{H-F} = 4.11 Hz, 1H), 7.21 (s, 1H), 7.18 (s, 1H), 7.14 (dd, J = 8.86, 2.66 Hz, 1H), 5.11 (m, 1H), 4.00 (s, 3H), 3.73-3.41 (m, 4H), 2.27 (m, 2H), 1.48 (s, 9H); ^{13}C NMR (126 MHz, CD_3OD): δ_C 156., 156.2, 155.6, 153.7, 147.3, 146.7, 124.4, 122.1 (d, J_{C-F} = 6.30 Hz), 121.9, 121.7 (d, J_{C-F} = 7.53 Hz), 120.7, 120.6, 116.4 (d, J_{C-F} = 21.39 Hz), 109.0, 108.6, 107.0, 106.1, 80.0, 77.6, 56.0, 50.8, 44.3, 43.8, 28.5; ^{19}F NMR (470 MHz, DMSO-d_6): δ_F -125.78; LRMS (ES$^+$) m/z 487.3 [M(^{35}Cl)+H]+ and 489.1 [M(^{37}Cl)+H]$^+$.

(R)-1-(3-((4-((3-Chloro-4-fluorophenyl)amino)-7-methoxyquinazolin-6-yl)oxy)pyrrolidin-1-yl)prop-2-en-1-one, 16

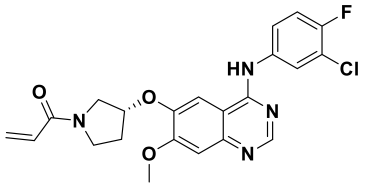

To a stirred solution of tert-butyl (R)-3-((4-((4-chloro-3-fluorophenyl)amino)-7-methoxyquinazolin-6-yl)oxy)pyrrolidine-1-carboxylate (144 mg, 0.29 mmol) in DCM (0.5 mL) was added 2,2,2-trifluoroacetic acid (168 mg, 1.47 mmol) in one portion and allowed to stir at room temperature for 1 h before passing through a SCX column and washing with 7 M NH_3 in MeOH (ca. 10 mL) and the resulting filtrate concentrated in vacuo to give a white solid. To this, a solution of acryloyl chloride (16 mg, 0.18 mmol) and sodium hydrogen carbonate (18 mg, 0.22 mmol) in THF (1 mL) was added under an atmosphere of N_2 and allowed to stir at 0 °C for 30 min then left to stir at room temperature overnight. After this time the reaction mixture was concentrated in vacuo and purified via flash column chromatography (eluent: 0-10% MeOH in DCM) to give (R)-1-(3-((4-((3-chloro-4-fluorophenyl)amino)-7-methoxyquinazolin-6-yl)oxy)pyrrolidine-1-yl)prop-2-en-1-one (17 mg, 0.04 mmol, 13%) as a white solid. R_f = 0.10 (10% MeOH in DCM); mp 211-214 ºC; $[\alpha]_D^{25.6}$ = +11.5º (c 1.13, EtOH); UV λ_{max} (nm) 331, 249, 208; IR: ν_{max}/cm^{-1} 3217 (m, N-H amide), 2925 (w, C-H aromatic), 1636 (m, C=O amide), 1575 (m, C=C alkene), 1495 (m, C=C aromatic); ^{1}H NMR (500 MHz, $CDCl_3$): δ_H 8.62 (s, 1H), 7.84 (dd, J_{H-F} = 6.60 Hz, J_{H-H} 2.60 Hz, 1H), 7.58 (ddd, J_{H-H} = 8.85, 2.60 Hz, J_{H-F} = 4.10 Hz, 1H), 7.46 (s, 1H), 7.28 (s, 1H), 7.20 (s, 1H), 6.48-6.37 (m, 2H), 6.32 (dd, J = 16.81, 1.86 Hz, 1H), 5.70 (dd, J = 16.81, 10.05 Hz, 1H), 5.08 (s, 1H), 3.97 (s, 3H), 3.92-3.70 (m, 6H); ^{13}C NMR (126 MHz, $CDCl_3$): δ_C 165.4, 156.9, 156.1, 153.8, 153.5, 148.4, 146.1, 128.9, 128.5, 125.3, 121.9 (d, J_{C-F} = 7.56 Hz), 120.7 (d, J_{C-F} = 18.6 Hz), 116.4 (d, J_{C-F} = 22.2 Hz), 109.4, 108.9, 108.4, 105.6, 76.3, 56.1, 51.9, 51.4, 44.9; ^{19}F NMR (470 MHz, $CDCl_3$): δ_F -121.14; LRMS (ES$^+$) m/z 443.3 196 [M(^{35}Cl)+H]$^+$ and 445.1 [M(^{37}Cl)+H]$^+$.

(S)-1-(3-((4-((3-Chloro-4-fluorophenyl)amino)-7-methoxyquinazolin-6-yl)oxy)pyrrolidin-1-yl)prop-2-en-1-one, 17

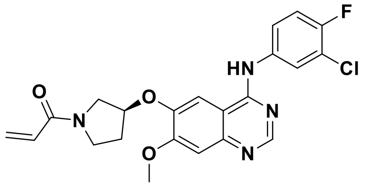

To a stirred solution of tert-butyl (S)-3-((4-((4-chloro-3-fluorophenyl)amino)-7-methoxyquinazolin-6-yl)oxy)pyrrolidine-1-carboxylate (63 mg, 0.13 mmol) in DCM (0.5 mL) was added 2,2,2-trifluoroacetic acid (74 mg, 0.65 mmol) in one portion and allowed to stir at room temperature for 1 h before passing through a SCX column and washing with 7 M NH_3 in MeOH (ca. 10 mL) and the resulting filtrate concentrated in vacuo to give a white solid. To this, a solution of acryloyl chloride (7 mg, 0.08 mmol) and sodium hydrogen carbonate (8 mg, 0.01 mmol) in THF (1 mL) was added under an atmosphere of N_2 and allowed to stir at 0 °C for 30 min then left to stir at room temperature overnight. After this time the reaction mixture was concentrated in vacuo and purified via flash column chromatography (eluent: 0-10% MeOH in DCM) to give (S)-1-(3-((4- ((3-chloro-4-fluorophenyl)amino)-7-methoxyquinazolin-6-yl)oxy)pyro-lidine-1-yl)prop2-en-1-one (17 mg, 0.04 mmol, 30%) as a white solid. R_f = 0.10 (10% MeOH in DCM); mp 211-214 ºC; $[\alpha]_D^{25.6}$ = -23.0º (c 0.30, EtOH); UV λ_{max} (nm) 331, 249, 208; IR: ν_{max}/cm^{-1} 3217 (m, N-H amide), 2925 (w, C-H aromatic), 1637 (m, C=O amide), 1575 (m, C=C alkene), 1495 (m, C=C aromatic); ^{1}H NMR (500 MHz, $CDCl_3$): δ_H 8.62 (s, 1H), 7.84 (dd, J_{H-F} = 6.60 Hz, J_{H-H} = 2.60 Hz, 1H), 7.58 (ddd, J_{H-H} = 8.85, 2.60 Hz, J_{H-F} = 4.11 Hz, 1H), 7.46 (s, 1H), 7.28 (s, 1H), 7.20 (s, 1H), 6.48-6.37 (m, 2H), 6.32 (dd, J = 16.81, 1.86 Hz, 1H), 5.70 (dd, J = 16.81, 10.05 Hz, 1H), 5.08 (s, 1H), 3.97 (s, 3H), 3.92-3.70 (m, 6H); ^{13}C NMR (126 MHz, $CDCl_3$): δ_C 165.4, 156.7, 156.2, 154.3, 154.0, 148.4, 146.0, 128.6, 128.2, 124.1, 121.7 (d, J_{C-F} = 7.60 Hz), 120.7 (d, J_{C-F} = 13.6 Hz), 116.4 (d, J_{C-F} = 22.7 Hz), 109.3, 108.9, 197 108.7, 105.6, 78.4, 56.0, 51.9, 51.1, 44.9; ^{19}F NMR (470 MHz, $CDCl_3$): δ_F -121.14; LRMS (ES$^+$) m/z 443.3 [M(^{35}Cl)+H]$^+$ and 445.1 [M(^{37}Cl)+H]$^+$.

tert-Butyl 3-((4-((3-chloro-4-fluorophenyl)amino)-7-methoxyquinazolin-6-yl)oxy)azetidine-1-carboxylate

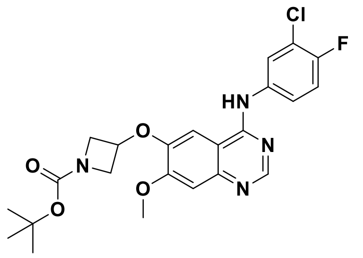

To a stirred solution of 4-((3-chloro-4-fluorophenyl)amino)-7-methoxyquinazolin-6-ol (200 mg, 0.63 mmol) in DMF (2.1 mL) was added potassium carbonate (173 mg, 1.25 mmol) and allowed to stir at room temperature for 1.5 h before addition of tert-butyl 3- ((methylsulfonyl)oxy)azetidine-1-carboxylate (173 mg, 0.69 mmol) and heated overnight at 80 °C. After this time, the reaction mixture was concentrated in vacuo and purification performed via flash column chromatography (eluent: 0-10% MeOH in DCM) to give tert-butyl 3-((4-((3-chloro-4-fluorophenyl)amino)-7-methoxyquinazolin-6- yl)oxy)azetidine-1-carboxylate (70 mg, 0.15 mmol, 24%) as a brown solid. 190 R_f = 0.16 (10% MeOH in DCM); mp 201-203 °C; UV λ_{max} (nm) 315, 251; IR: ν_{max}/cm^{-1} 3371 (m, N-H), 2964 (m, C-H aromatic), 1673 (s, C=O carbamate), 1497 (s, C=C aromatic); ^{1}H NMR (500 MHz, $CDCl_3$): δ_H 8.66 (s, 1H), 7.85 (dd, J_{H-F} = 6.50 Hz, J_{H-H} = 2.68 Hz, 1H), 7.51 (ddd, J_{H-H} = 8.89, 2.72 Hz, J_{H-F} = 4.05 Hz, 1H), 7.31 (s, 1H), 7.29 (s, 1H), 7.17 (d, J = 8.89 Hz, 1H), 6.89 (s, 1H), 5.05 (tt, J = 6.64, 4.31 Hz, 1H), 4.37 (dd, J = 9.89, 6.62 Hz, 2H), 4.16 (dd, J = 9.87, 4.31 Hz, 2H), 4.02 (s, 3H), 1.45 (s, 9H); ^{13}C NMR (126 MHz, $CDCl_3$): δ_C 156.3, 156.2, 155.2, 154.0, 148.3, 146.5, 124.3, 121.8 (d, J_{C-F} = 6.54 Hz), 121.8, 121.2, 117.9, 116.7 (d, J_{C-F} = 22.5 Hz), 116.6, 108.8, 102.5, 80.3, 67.3, 58.3, 56.3, 28.4; ^{19}F NMR (470 MHz, $CDCl_3$): δ_F -120.56; LRMS (ES$^+$) m/z 475.3 [M(^{35}Cl)+H]$^+$ and 477.3 [M(^{37}Cl)+H]$^+$.

1-(3-((4-((3-Chloro-4-fluorophenyl)amino)-7-methoxyquinazolin-6- yl)oxy)azetidin-1-yl)prop-2-en-1-one, 18

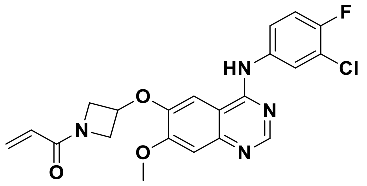

To a stirred solution of tert-butyl 3-((4-(3-chloro-4-fluorophenyl)amino)-7- methoxyquinazolin-6-yl)azetidine-1-carboxylate (33 mg, 0.14 mmol) in DCM (0.5 mL) was added 2,2,2-trifluoroacetic acid (100 µL, 0.86 mmol) in one portion and allowed to stir at room temperature for 1 h before passing through a SCX column and washing with 7 M NH3 in MeOH (ca. 10 mL) and the resulting filtrate concentrated in vacuo to give a white solid. To this, a prepared solution of acryloyl chloride (17 mg, 0.19 mmol) in THF (1 mL) was added followed by and sodium hydrogen carbonate (19 mg, 0.23 mmol) under an atmosphere of N_2 and allowed to stir at 0 °C for 30 min then left to stir at room temperature overnight. After this time, the reaction mixture was concentrated in vacuo and purified was performed via flash column chromatography (eluent: 0-10% MeOH in DCM) to give 1-(3-((4-((3-chloro-4-fluorophenyl)amino)-7-methoxyquinazolin-6-yl)oxy)azetidin-1-yl)prop-2-en-1-one (24 mg, 0.06 mmol, 42%) as a white solid. R_f = 0.14 (10% MeOH in DCM); mp 200-202 ºC; UV λ_{max} (nm) 321, 249; IR: ν_{max}/cm^{-1} 3306 (w, N-H amide), 2916 (w, C-H aromatic), 1644 (m, C=O amide), 1574 (m, C=C alkene), 1500 (m, C=C aromatic); ^{1}H NMR (500 MHz, $CDCl_3$): δ_H 8.64 (s, 1H), 8.57 (s, 1H), 7.83 (dd, $J_{H\text{-}F}$ = 6.58 Hz, $J_{H\text{-}H}$ 2.61 Hz, 1H), 7.56 (ddd, $J_{H\text{-}H}$ = 8.84, 2.73 Hz, $J_{H\text{-}F}$ = 4.02 Hz, 1H), 7.35 (s, 1H), 7.28 (s, 1H), 7.12 (d, *J* = 8.78 Hz, 1H), 6.32-6.19 (m, 2H), 5.70 (dd, *J* = 9.71, 2.17 Hz, 1H), 5.14 (tt, *J* = 6.60, 4.48 Hz, 1H), 4.75-4.61 (m, 2H), 4.52- 4.22 (m, 2H), 4.02 (s, 3H); ^{13}C NMR (126 MHz, CD_3OD): δ_C 166.4, 157.2, 155.8, 153.0, 147.1, 146.6, 135.9, 127.1, 125.7, 124.7, 122.8 (d, $J_{C\text{-}F}$ = 7.56 Hz), 122.5, 120.1, 116.0 (d, $J_{C\text{-}F}$ = 21.4 Hz), 108.8, 106.5, 103.5, 66.9, 57.2, 55.3; ^{19}F NMR (470 MHz, $CDCl_3$): δ_F -121.11; LRMS (ES$^+$) m/z 429.3 [M(^{35}Cl)+H]$^+$ and 431.3 [M(^{37}Cl)+H]$^+$.

(*R*)-1-(3-((4-((3-Chloro-4-fluorophenyl)amino)-7-methoxyquinazolin-6-yl)oxy)pyrrolidin-1-yl)prop-2-yn-1-one, 19

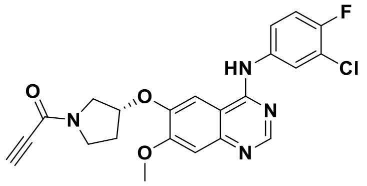

To a stirred solution of tert-butyl (R)-3-((4-((4-chloro-3-fluorophenyl)amino)-7-methoxyquinazolin-6-yl)oxy)pyrrolidine-1-carboxylate (144 mg, 0.29 mmol) in DCM (0.5 mL) was added 2,2,2-trifluoroacetic acid (168 mg, 1.47 mmol) in one portion and allowed to stir at room temperature for 1 h before passing through a SCX column and washing with 7 M NH_3 in MeOH (ca. 10 mL) and the resulting filtrate concentrated in vacuo to give a white solid. To this was added a solution of HATU (110 mg, 0.29 mmol), DMAP (14 mg, 0.12 mmol) and propiolic acid (20 mg, 0.29 mmol) in DMF (10 mL) under an atmosphere of N_2 and allowed to stir at room temperature for 2 h before being poured over water (ca. 30 mL). The resulting precipitate was filtered and dried in vacuo to give (R)-1-(3-((4-((3-chloro-4-fluorophenyl)amino)-7-methoxyquinazolin-6-yl)oxy)pyrrolidin-1-yl)prop-2-yn-1-one (97 mg, 0.22 mmol, 76%) as a yellow solid. R_f = 0.12 (20% MeOH in DCM); mp 201-204 ºC; $[\alpha]_D^{25.6}$ = +13.3º (*c* 0.15, EtOH); UV λ_{max} (nm) 330, 247; IR: ν_{max}/cm^{-1} 3065 (w, N-H amide), 2958 (w, C-H alkene), 2916 (w, C-H aromatic), 2826 (w, C-H alkane), 2095 (m, C≡C alkyne), 1607 (m, C=O amide), 1579 (m, C=C alkene), 1495 (m, C=C aromatic); ^{1}H NMR (500 MHz, $CDCl_3$): δ_H 8.66 (s, 1H), 7.91 (dd, $J_{H\text{-}F}$ = 6.61 Hz, $J_{H\text{-}H}$ = 2.70 Hz, 1H), 7.58 (ddd, $J_{H\text{-}H}$ = 8.80, 2.60 Hz, $J_{H\text{-}F}$ = 4.28 Hz 1H), 7.46 (s, 1H), 7.34 (s, 1H), 7.29 (s, 1H), 7.15 (d, *J* = 8.80 Hz, 1H). 5.19 (s, 1H), 3.98 (s, 1H), 3.97 (s, 3H), 3.73 (m, 4H), 3.10 (m, 2H); ^{13}C NMR (126 MHz, $CDCl_3$): δ_C 160.1, 156.8, 153.9, 153.8, 146.6, 124.5, 124.1, 122.2, 121.6 (d, $J_{C\text{-}F}$ = 7.61 Hz), 120.9, 120.8, 117.1 (d, $J_{C\text{-}F}$ = 17.61 Hz), 116.5, 108.7, 105.4, 85.7, 81.1, 78.2, 56.3, 51.0, 46.5, 43.8; ^{19}F NMR (470 MHz, $CDCl_3$): δ_F -120.73; LRMS (ES$^+$) *m/z* 441.3 [M(^{35}Cl)+H]$^+$ and 443.1 [M(^{37}Cl)+H]$^+$.

***S*)-1-(3-((4-((3-Chloro-4-fluorophenyl)amino)-7-methoxyquinazolin-6-yl)oxy)pyrrolidin-1-yl)prop-2-yn-1-one, 20**

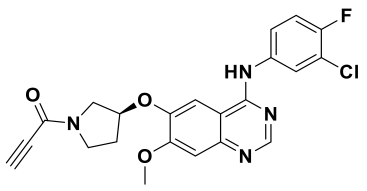

To a stirred solution of tert-butyl (S)-3-((4-((4-chloro-3-fluorophenyl)amino)-7-methoxyquinazolin-6-yl)oxy)pyrrolidine-1-carboxylate (140 mg, 0.29 mmol) in DCM (0.5 mL) was added 2,2,2-trifluoroacetic acid (163 mg, 1.43 mmol) in one portion and allowed to stir at room temperature for 1 h before passing through a SCX column and washing with 7 M NH_3 in MeOH (ca. 10 mL) and the resulting filtrate concentrated in vacuo to give a white solid. To this was added HATU (110 mg, 0.29 mmol), DMAP (14 mg, 0.12 mmol) and propiolic acid (20 mg, 0.29 mmol) in DMF (10 mL) under an atmosphere of N_2 and allowed to stir at room temperature for 2 h before being poured over water (ca. 30 mL). The resulting precipitate was filtered and dried in vacuo to give (S)-1-(3-((4-((3-chloro-4-fluorophenyl)amino)-7-methoxyquinazolin-6-yl)oxy)pyrrolidin-1-yl)prop-2-yn-1-one (93 mg, 0.21 mmol, 72%) as a yellow solid. R_f = 0.12 (20% MeOH in DCM); mp 201-204 ºC; $[\alpha]_D^{25.6}$ = -5.4º (*c* 0.37, EtOH); UV λ_{max} (nm) 330, 247; IR: ν_{max}/cm^{-1} 3065 (w, N-H amide), 2958 (w, C-H alkene), 2916 (w, C-H aromatic), 2826 (w, C-H alkane), 2095 (m, C≡C alkyne), 1607 (m, C=O amide), 1579 (m, C=C alkene), 1495 (m, C=C aromatic); ^{1}H NMR (500 MHz, $CDCl_3$): δ_H 8.66 (s, 1H), 7.91 (dd, J_{H-F} = 6.61 Hz, J_{H-H} 2.70 Hz, 1H), 7.58 (ddd, J_{H-H} = 8.80, 2.60 Hz, J_{H-F} = 4.28 Hz 1H), 7.46 (s, 1H, H^6), 7.34 (s, 1H, H^3), 7.29 (s, 1H), 7.15 (d, J = 8.80 Hz, 1H). 5.19 (s, 1H), 3.98 (s, 1H), 3.97 (s, 3H), 3.73 (m, 4H), 3.10 (m, 2H); ^{13}C NMR (126 MHz, $CDCl_3$): δ_C 160.2, 156.8, 154.0, 153.8, 146.7, 124.5, 124.0, 122.2, 121.6 (d, J_{C-F} = 7.58 Hz), 120.9, 120.7, 117.1 (d, J_{C-F} = 17.64 Hz), 116.5, 108.7, 105.8, 85.8, 81.2, 78.2, 56.3, 50.9, 46.5, 43.9; ^{19}F NMR (470 MHz, $CDCl_3$): δ_F -120.73 (s, 1F, F^{18}); LRMS (ES$^+$) *m/z* 441.3 [M(^{35}Cl)+H]$^+$ and 443.1 [M(^{37}Cl)+H]$^+$.

1-(3-((4-((3-Chloro-4-fluorophenyl)amino)-7-methoxyquinazolin-6-yl)oxy)azetidin-1-yl)prop-2-yn-1-one, 21

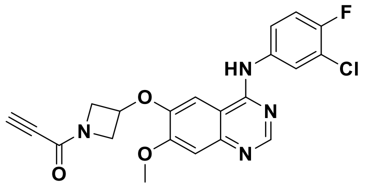

To a stirred solution of tert-butyl 3-((4-(3-chloro-4-fluorophenyl)amino)-7-methoxyquinazolin-6-yl)azetidine-1-carboxylate (52 mg, 0.22 mmol) in DCM (0.5 mL) was added 2,2,2-trifluoroacetic acid (100 mg, 0.86 mmol) in one portion and allowed to stir at room temperature for 1 h before passing through a SCX column and washing with 7 M NH_3 in MeOH (ca. 10 mL) and the resulting filtrate concentrated in vacuo to give a white solid. To this, a solution of DIPEA (110 µL, 0.85 mmol), HATU (97 mg, 0.26 mmol), and propiolic acid (16 mg, 0.23 mmol) in DMA (1.0 mL) was added under an atmosphere of N_2 and allowed to stir at room temperature for 2 h before being poured over water (ca. 30 mL). The resulting precipitate was filtered and dried in vacuo to give 1-(3-((4-((3-chloro-4-fluorophenyl)amino)-7-methoxyquinazolin-6-yl)oxy)azetidin-1-yl)prop-2-yn-1-one (55 mg, 0.13 mmol, 60%) as a yellow solid. R_f = 0.18 (20% MeOH in DCM); mp 208-210 ºC; UV λ_{max} (nm) 304, 237; IR: ν_{max}/cm^{-1} 3267 (w, N-H amide), 2936 (m, C-H aromatic), 1574 (m, C=C alkene), 1527 (m, C=C aromatic), carbonyl stretches not visualised; ^{1}H NMR (500 MHz, $CDCl_3$): δ_H 8.63 (s, 1H), 8.62 (s, 1H), 8.06 (dd, J_{H-F} = 6.53 Hz, J_{H-H} 2.47 Hz, 1H), 7.89 (dd, J = 8.96, 2.59 Hz, 1H), 7.63 (dd, J_{H-H} = 8.94 Hz, J_{H-F} = 4.14 Hz, 1H), 7.26 (s, 1H), 7.19 (s, 1H), 5.20 (tt, J = 10.51, 4.71 Hz, 1H), 4.92 (dd, J = 10.42, 6.67 Hz, 2H), 4.44 (dd, J = 10.11, 4.71 Hz, 2H), 3.99 (s, 3H), 3.02 (s, 1H); ^{13}C NMR (126 MHz, CD_3OD): δ_C 158.9, 157.2, 155.5, 153.8, 147.2, 146.4, 135.9, 124.7 (d, J_{C-F} = 8.82 Hz), 122.8, 122.7, 120.1, 116.0 (d, J_{C-F} = 22.68 Hz), 108.8, 107.0, 106.5, 106.0, 103.9, 67.5, 60.5, 38.7; ^{19}F NMR (470 MHz, $CDCl_3$): δ_F -121.62; LRMS (ES$^+$) *m/z* 427.3 [M(^{35}Cl)+H]$^+$ and 429.3 [M(^{37}Cl)+H]$^+$.

(R)-N-(3-chloro-4-fluorophenyl)-7-methoxy-6-(pyrrolidin-3-yloxy)quinazolin-4-amine, 22

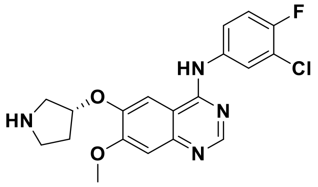

To a stirred solution of tert-butyl (R)-3-((4-((4-chloro-3-fluorophenyl)amino)-7-methoxyquinazolin-6-yl)oxy)pyrrolidine-1-carboxylate (463 mg, 0.95 mmol) in DCM (1.9 mL) was added 2,2,2-trifluoroacetic acid (362 mg, 4.72 mmol) in one portion and allowed to stir at room temperature for 1 h before passing through a SCX column and washing with 7 M NH_3 in MeOH (ca. 10 mL) and the resulting filtrate concentrated in vacuo to give a white solid.

(R)-1-(3-((4-((3-chloro-4-fluorophenyl)amino)-7-methoxyquinazolin-6-yl)oxy)pyrrolidin-1-yl)ethan-1-one, 22

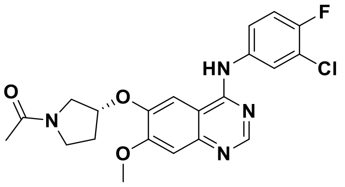

To a stirred solution of (R)-N-(3-chloro-4-fluorophenyl)-7-methoxy-6-(pyrrolidin-3-yloxy)quinazolin-4-amine (100 mg, 0.26 mmol) in THF (2.0 mL) was added acetyl chloride (11 µL, 0.156 mmol) and sodium hydrogen carbonate (16 mg, 0.026 mmol) in THF (1 mL) and allowed to stir at room temperature for 30 min then left to stir at room temperature overnight. After this time the reaction mixture was concentrated in vacuo and purified via flash column chromatography (eluent: 0-10% MeOH in DCM) to give (R)-1-(3-((4-((3-chloro-4-fluorophenyl)amino)-7-methoxyquinazolin-6-yl)oxy)pyrrolidin-1-yl)ethan-1-one (19.1 mg, 45.3 µmol, 17%) as a white solid.

R_f = 0.56 (10% MeOH in DCM); mp 201-204 ºC; $[\alpha]_D^{23.6}$ = +24.0º (*c* 0.25, EtOH); UV λ_{max} (nm) 253, 224; IR: ν_{max}/cm^{-} 3293 (br, N-H), 2952 (w, C-H aromatic), 1618 (s, C=O amide), 1576 (m, C=C aromatic), 1499 (m, C=C aromatic); ^{1}H NMR (500 MHz, 393K, DMSO): δ_H 8.52 (s, 1H), 8.08 (dd, *J* = 6.7, 2.7 Hz, 1H), 7.90 (s, 1H), 7.81 (ddd, *J* = 9.0, 4.3, 2.6 Hz, 1H), 7.36 (t, *J* = 9.0 Hz, 1H), 7.27 (s, 1H), 5.20 (s, 1H), 3.98 (s, 3H), 3.70 – 3.53 (m, 4H), 2.25 (s, 2H), 2.00 (s, 3H); ^{13}C NMR (126 MHz, 393K, DMSO): δ_C 179.67, 168.66, 157.07, 156.51, 155.07, 153.40, 153.13, 147.70 (d, *J* = 95.4 Hz), 141.23, 137.44, 124.45, 123.11 (d, *J* = 6.4 Hz), 119.52 (d, *J* = 18.2 Hz), 116.72 (d, *J* = 21.8 Hz), 109.67, 108.91, 108.03, 76.19, 56.67, 50.21, 45.26, 32.09, 22.29; ^{19}F NMR (470 MHz, $CDCl_3$): δ_F -122.81; LRMS (ES$^+$) *m/z* 431.3 [M(^{35}Cl)+H]$^+$ and 433.3 [M(^{37}Cl)+H]$^+$.

(S)-N-(3-chloro-4-fluorophenyl)-7-methoxy-6-(pyrrolidin-3-yloxy)quinazolin-4-amine, 22

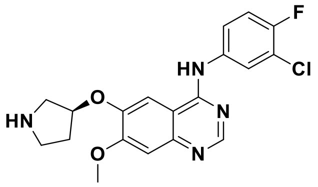

To a stirred solution of tert-butyl (s)-3-((4-((4-chloro-3-fluorophenyl)amino)-7-methoxyquinazolin-6-yl)oxy)pyrrolidine-1-carboxylate (499 mg, 1.02 mmol) in DCM (2.0 mL) was added 2,2,2-trifluoroacetic acid (389 mg, 5.09 mmol) in one portion and allowed to stir at room temperature for 1 h before passing through a

SCX column and washing with 7 M NH_3 in MeOH (ca. 10 mL) and the resulting filtrate concentrated in vacuo to give a white solid.

(S)-1-(3-((4-((3-chloro-4-fluorophenyl)amino)-7-methoxyquinazolin-6-yl)oxy)pyrrolidin-1-yl)ethan-1-one, 23

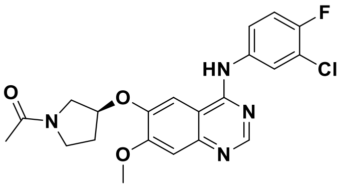

To a stirred solution of (S)-N-(3-chloro-4-fluorophenyl)-7-methoxy-6-(pyrrolidin-3-yloxy)quinazolin-4-amine (100 mg, 0.26 mmol) in THF (2.0 mL) was added acetyl chloride (11 µL, 0.156 mmol) and sodium hydrogen carbonate (16 mg, 0.026 mmol) in THF (1 mL) and allowed to stir at room temperature for 30 min then left to stir at room temperature overnight. After this time the reaction mixture was concentrated in vacuo and purified via flash column chromatography (eluent: 0-10% MeOH in DCM) to give (S)-1-(3-((4-((3-chloro-4-fluorophenyl)amino)-7-methoxyquinazolin-6-yl)oxy)pyrrolidin-1-yl)ethan-1-one (23.8 mg, 55.0 µmol, 21%) as a white solid.

R_f = 0.56 (10% MeOH in DCM); mp 209-212 ºC; $[\alpha]_D^{23.4}$ = -23.4º (*c* 0.47, EtOH); UV λ_{max} (nm) 221; IR: ν_{max}/cm^{-} 3316 (br, N-H), 2950 (w, C-H aromatic), 1617 (s, C=O amide), 1576 (m, C=C aromatic), 1495 (m, C=C aromatic); ^{1}H NMR (500 MHz, d^6-DMSO): δ_H 9.26 (s, 1H), 8.51 (s, 1H), 8.08 (dd, *J* = 6.8, 2.6 Hz, 1H), 7.81 (ddd, *J* = 8.9, 4.3, 2.6 Hz, 1H), 7.35 (t, *J* = 9.0 Hz, 1H), 7.27 (s, 1H), 5.20 (s, 1H), 3.98 (s, 3H), 3.75 – 3.61 (m, 4H), 2.25 (s, 2H), 2.00 (s, 3H); ^{13}C NMR (126 MHz, 393K, DMSO): δ_C 172.80, 168.65, 157.06, 156.49, 155.06, 153.43, 153.12, 147.76 (d, *J* = 113.5 Hz), 137.46, 124.43, 123.08 (d, *J* = 6.8 Hz), 119.52 (d, *J* = 18.2 Hz), 116.70 (d, *J* = 21.8 Hz), 109.69, 108.96, 108.12, 74.34, 56.66, 51.32, 45.26, 31.99, 22.30; ^{19}F NMR (470 MHz, d^6-DMSO): δ_F -122.87; LRMS (ES$^+$) *m/z* 431.3 $[M(^{35}Cl)+H]^+$ and 433.3 $[M(^{37}Cl)+H]^+$.

NMR and HPLC Spectra for key compounds

N-(4-(4-(3-((4-((3-chloro-4-fluorophenyl)amino)-7-methoxyquinazolin-6-yl)oxy)propyl)piperazin-1-yl)-4-oxobutyl)-3-(5,5-difluoro-7,9-dimethyl-5H-5l4,6l4-dipyrrolo[1,2-c:2',1'-f][1,3,2]diazaborinin-3-yl)propenamide, 6

Acidic HPLC – 100%

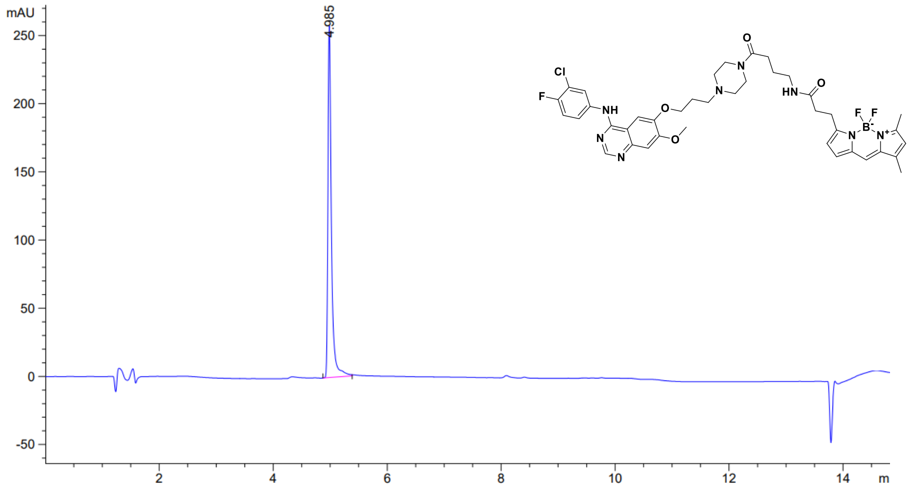

Basic HPLC – 100%

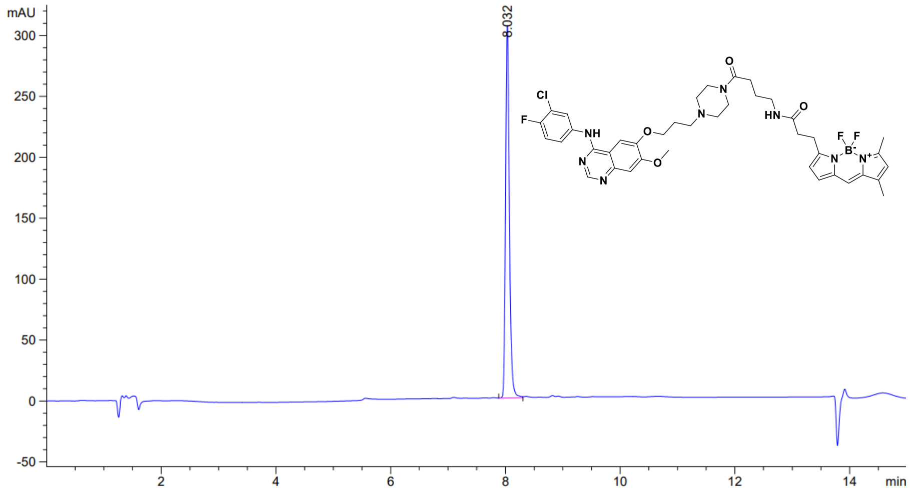

Proton NMR

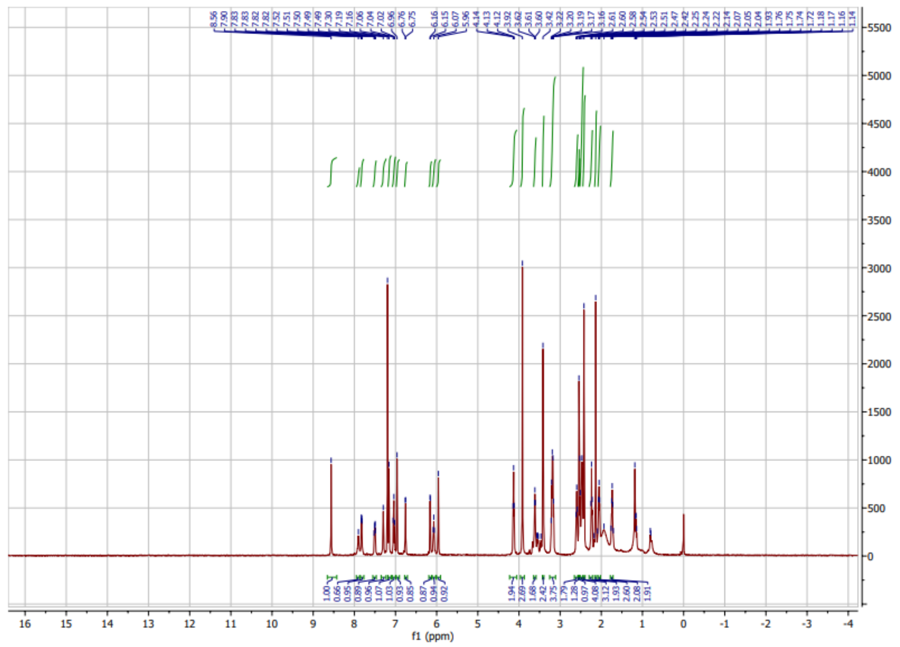

Carbon NMR

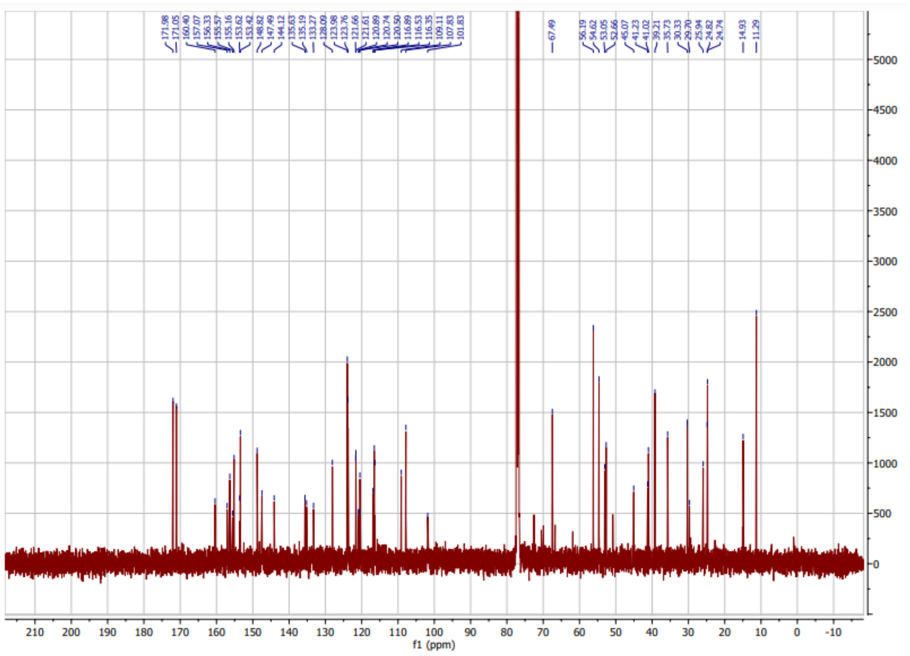

Fluorine NMR

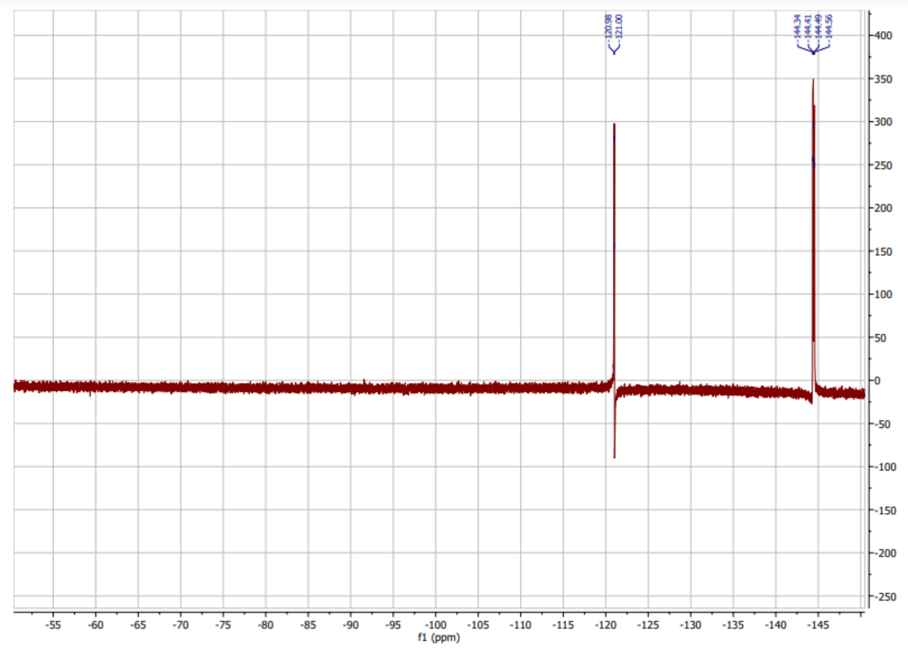

(R)-1-(3-((4-((3-chloro-4-fluorophenyl)amino)-7-methoxyquinazolin-6-yl)oxy)pyrrolidin-1-yl)prop-2-en-1-one, 16

Acidic HPLC – 98%

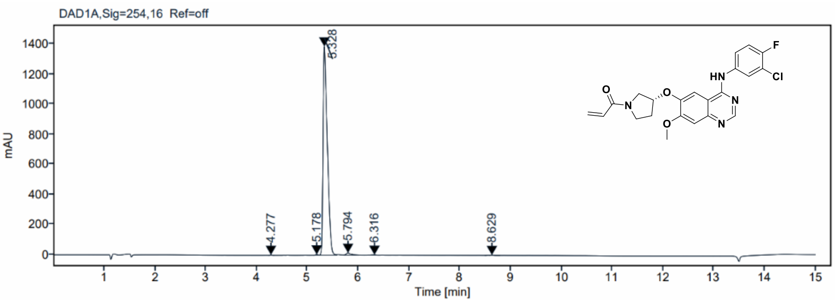

Basic HPLC – 97%

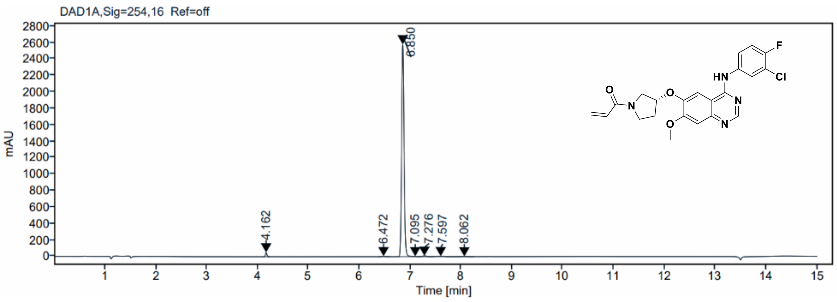

Proton NMR

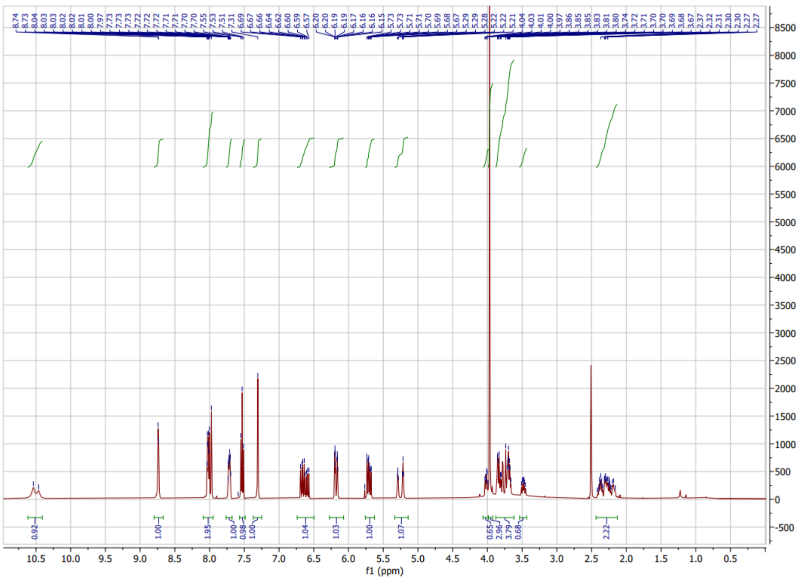

Carbon NMR

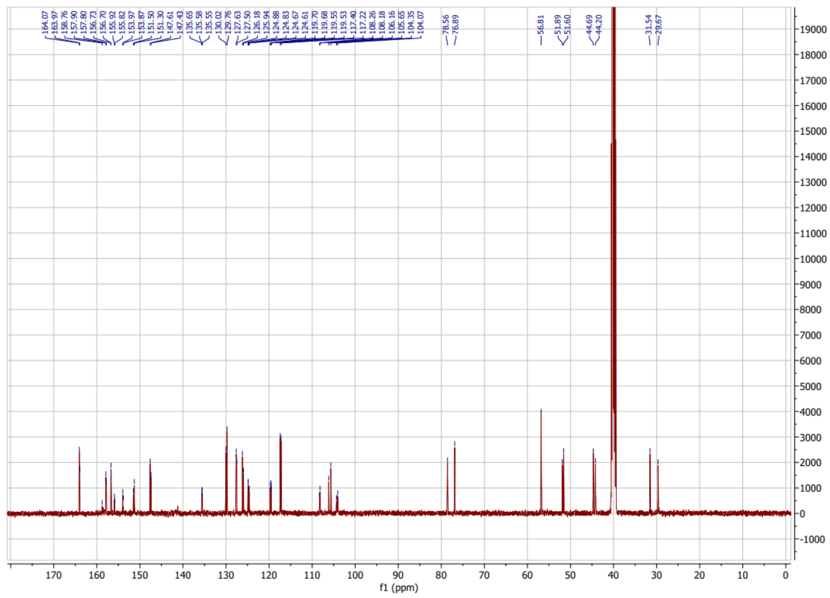

Fluorine NMR

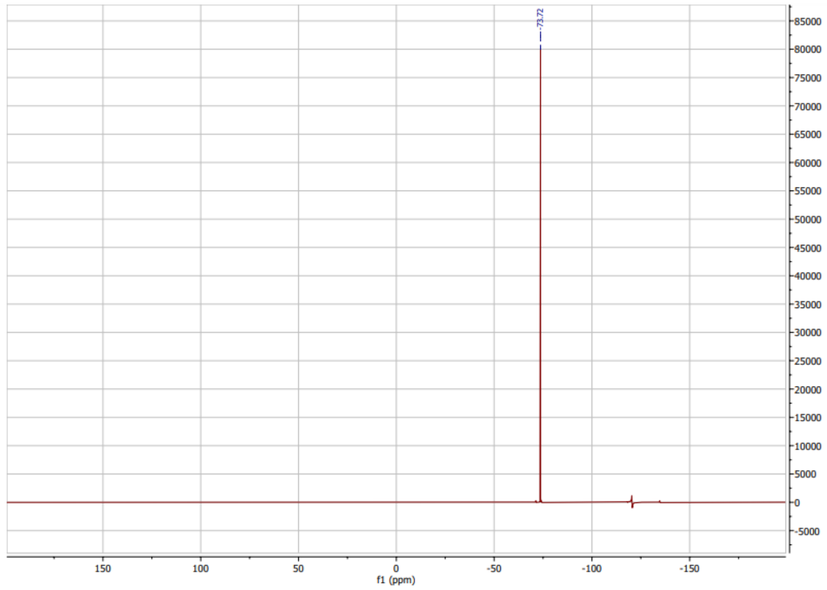

(S)-1-(3-((4-((3-chloro-4-fluorophenyl)amino)-7-methoxyquinazolin-6-yl)oxy)pyrrolidin-1-yl)prop-2-en-1-one, 17

Acidic HPLC – 97%

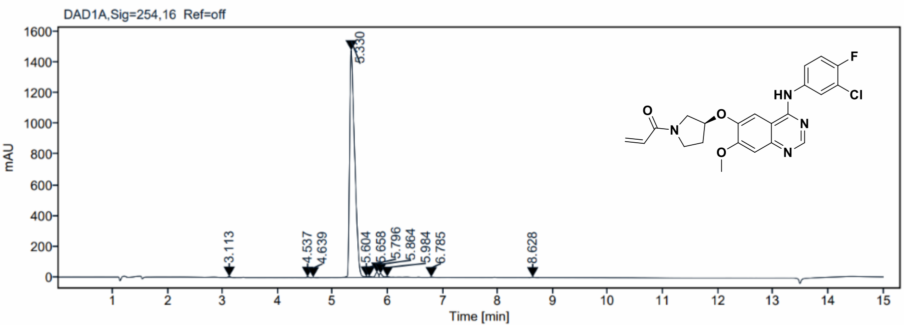

Basic HPLC – 97%

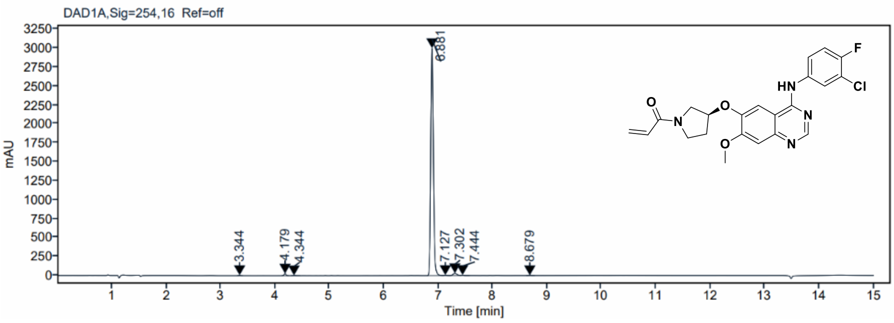

Proton NMR

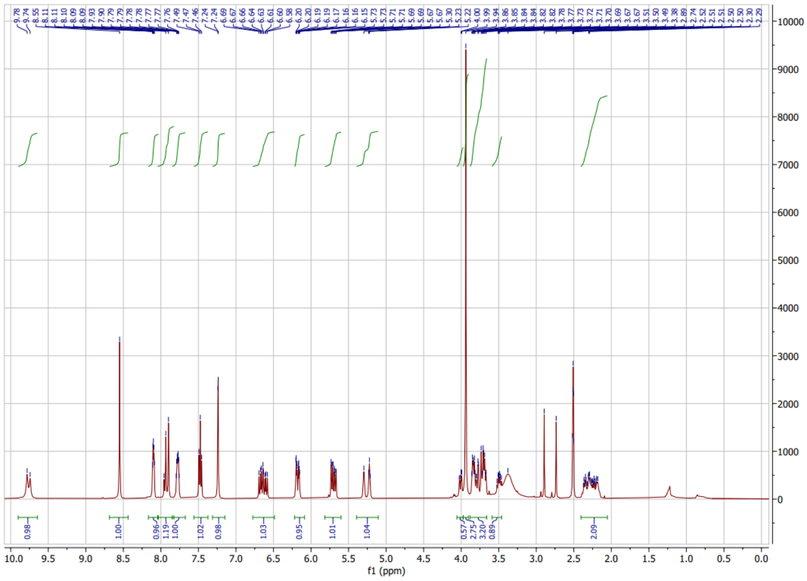

Carbon NMR

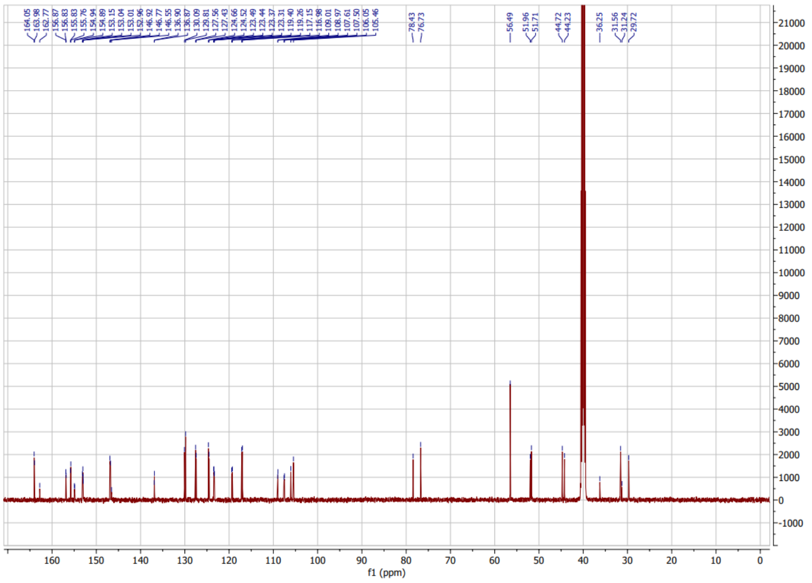

Fluorine NMR

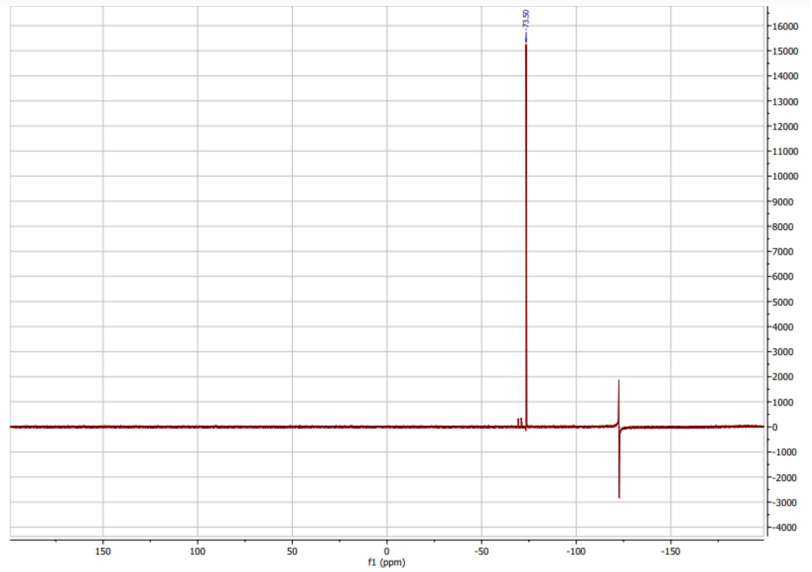

Intact Protein Mass Spectrum

1-(4-((4-((3,4-Dichloro-2-fluorophenyl)amino)-7-methoxyquinazolin-6-yl)oxy)piperidin-1-yl)prop-2-en-1-one, 4

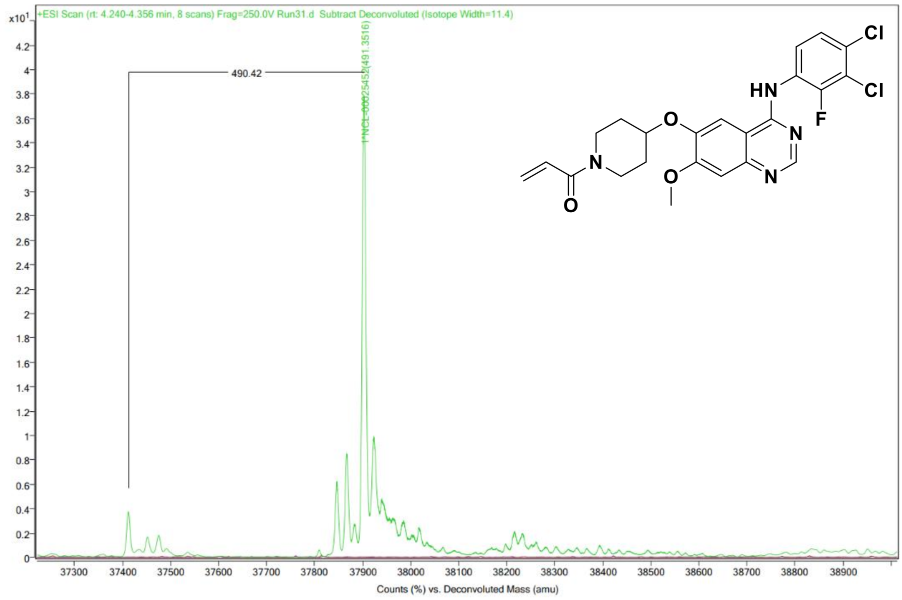

1-(4-((4-((3-Chloro-2-fluorophenyl)amino)quinazolin-6-yl)oxy)piperidin-1- yl)prop-2-en-1-one, 7

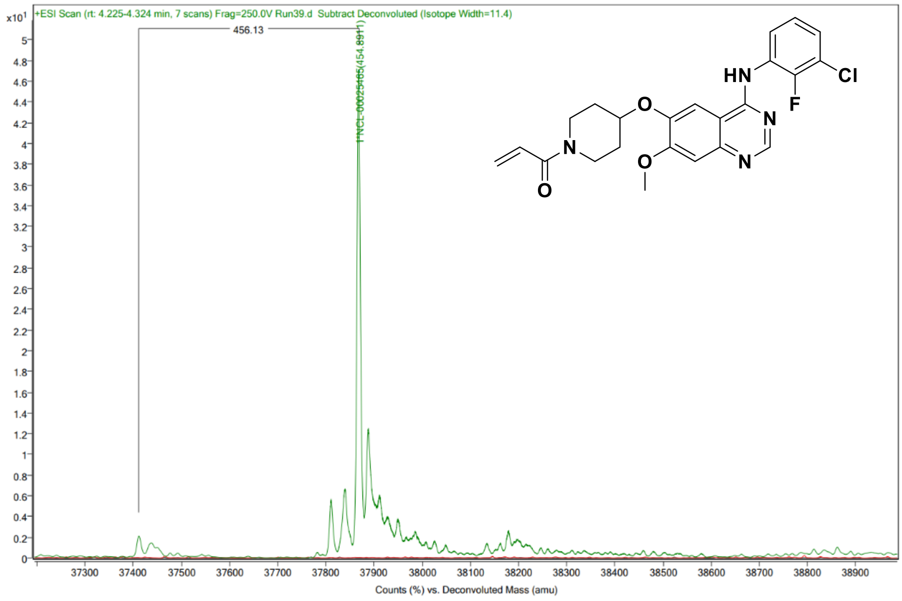

1-(4-((4-((3-Chloro-4-fluorophenyl)amino)-7-methoxyquinazolin-6-yl)oxy)piperidin-1-yl)prop-2-en-1-one, 8

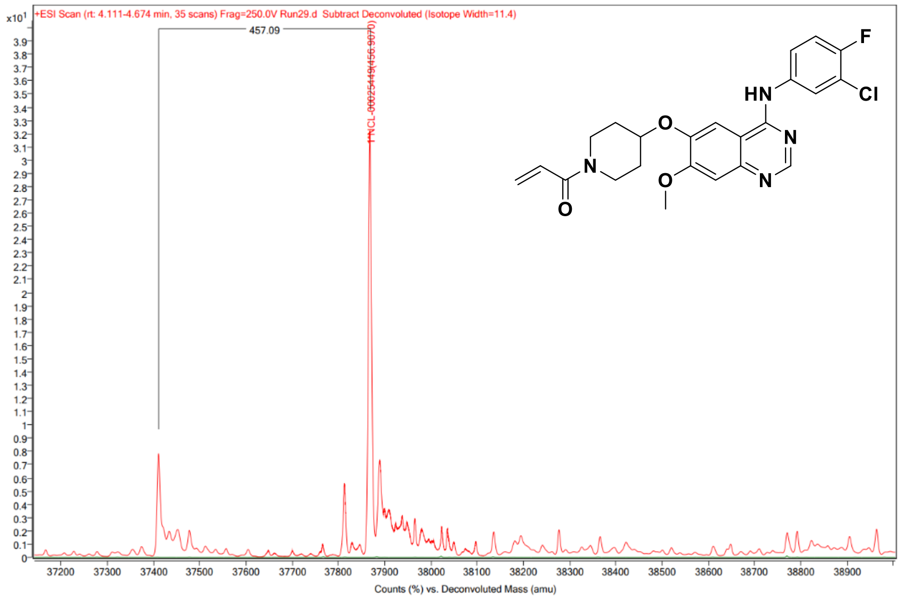

1-(4-((4-((5-Chloro-2-fluorophenyl)amino)-7-methoxyquinazolin-6-yl)oxy)piperidin-1-yl)prop-2-en-1-one, 9

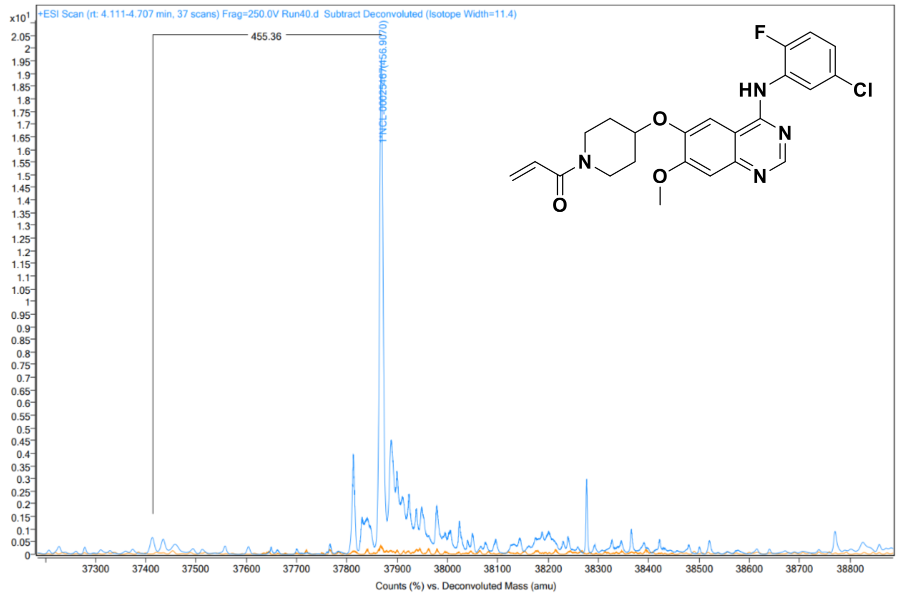

1-(4-((4-((3-Chloro-2-fluorophenyl)amino)quinazolin-6-yl)oxy)piperidin-1-yl)prop-2-en-1-one, 14

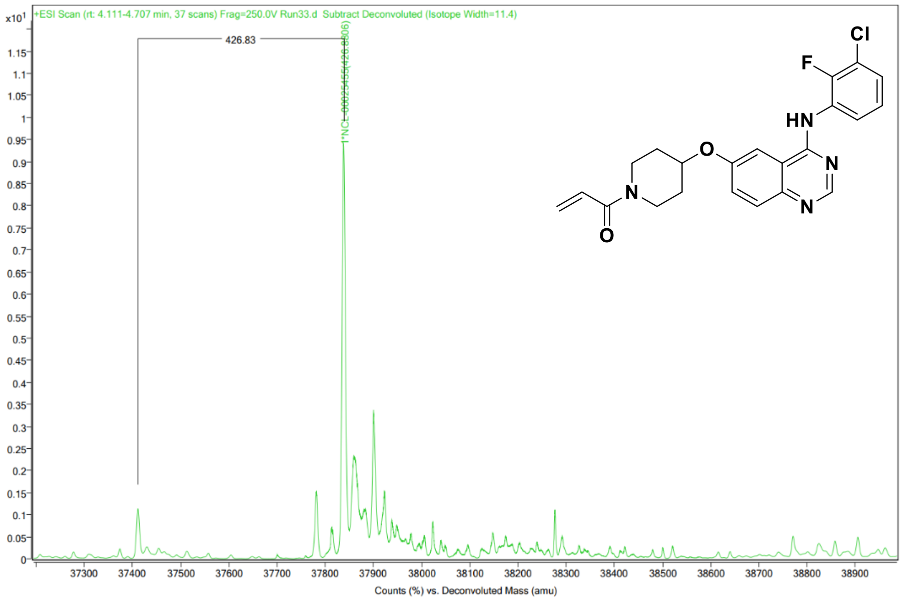

(R)-1-(3-((4-((3-chloro-4-fluorophenyl)amino)-7-methoxyquinazolin-6-yl)oxy)pyrrolidin-1-yl)prop-2-en-1-one, 16

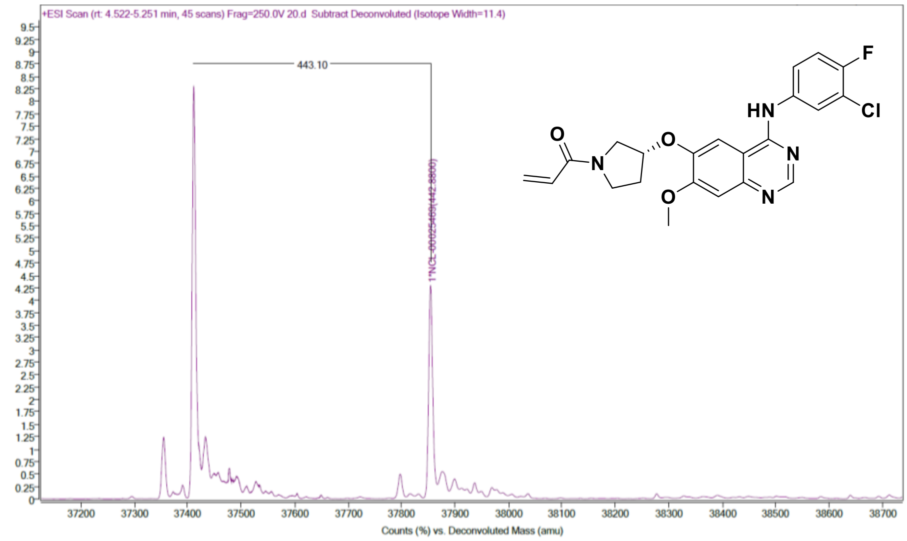

(*S*)-1-(3-((4-((3-chloro-4-fluorophenyl)amino)-7-methoxyquinazolin-6-yl)oxy)pyrrolidin-1-yl)prop-2-en-1-one, 17

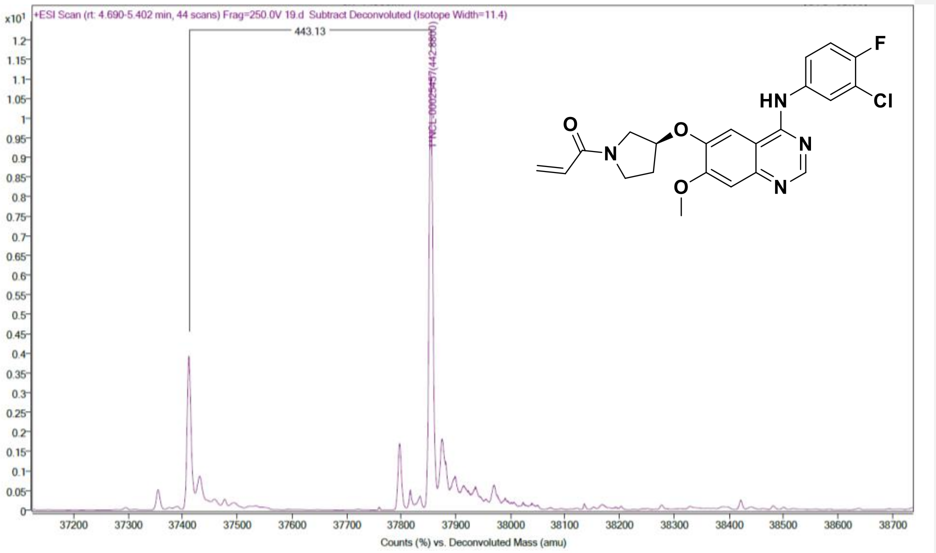

1-(3-((4-((3-Chloro-4-fluorophenyl)amino)-7-methoxyquinazolin-6- yl)oxy)azetidin-1-yl)prop-2-en-1-one, 18

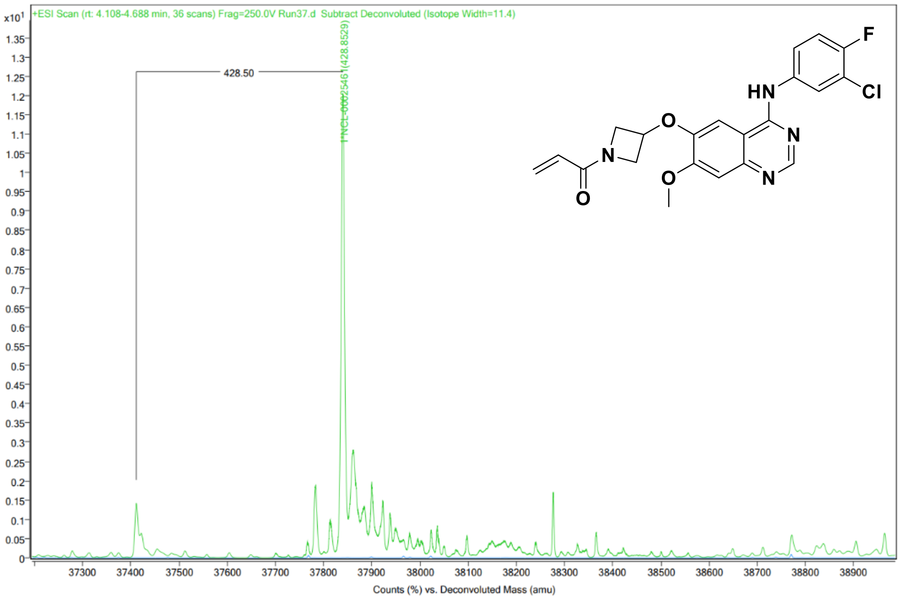

Computational Methods

1.1 Molecular docking

To understand the difference in activity of both compounds, we first modelled the interactions of both isomers with EGFR in SeeSAR (v. 13.2),[8] starting from the crystal structures of both compounds (reactants), estimating the binding affinities. To examine the adduct of less active isomer (**16**), we employed covalent docking (SeeSAR 13.2),[8] using 300 maximum poses, ring puckering enabled, and high clash tolerance. We applied the same procedure to the more active compound (**17**), which had crystal structure of the adduct solved, as a positive control. The binding affinities have been estimated using HYDE[9] scoring function, which calculates the intrinsic (electrostatic and van der Waals) and desolvation terms for each heavy atom of the ligand and the receptor counterparts.

1.2 Molecular dynamics (MD) simulations

To investigate plausible origins of affinity differences we carried out equilibrium all-atom MD simulations of **16** and **17** in water.

In the setup, we have used three structures: **17**-EGFR (conformer **A**, crystal structure), which was reproduced by the covalent docking, **16**-EGFR (conformer **B**, non-covalent complex; starting from the crystal structure), and **16**-EGFR adduct (conformer **C**), generated by covalent docking.

Preparation of the complexes for MD simulations has been done using UCSF Chimera.[10] To prepare experimental structures (corresponding to **A** and **B**), we removed all water molecules, non-complexed ions and crystallisation additives; added missing hydrogen atoms and any missing loops in via MODELLER interface[11] and modelled any incomplete side chains using Dunbrack rotamer library.[12] Side chains with alternate location were fixed by selecting the highest-occupancy conformers. Ligands have been parametrised using ACPYPE,[13] with GAFF force field[14] and AM1-BCC[15] partial atomic charges assigned.

All simulations were performed using Gromacs 2023.[16] EGFR was parametrised using the AMBER99SB-ILDN force field, with the TIP3P water model.[17] Box distance was set to 1 nm and periodic boundary conditions were applied. The structural parameters for the ligand-cysteine adduct were extracted from GAFF. The box was solvated and Na^+ and Cl^- ions were added to achieve a 0.1 M concentration and to maintain unit neutrality. The solvated systems were energy minimised and equilibrated. The minimisation ran using steepest descent for 1,000 cycles followed by the conjugate gradient. Energy step size was set to 0.001 nm and the maximum number of steps was set to 50,000. The minimisation was stopped when the maximum force fell below 1000 kJ/mol/nm using the Verlet cut-off scheme. Treatment of long-range electrostatic interactions was set to Particle Mesh-Ewald (PME),[18] and the short-range electrostatic and van der Waals cut-off set to 1.0 nm. After the energy minimization, heating to 300 K was performed for 20 ps with a time step of 2 fs and position restraints applied to the backbone in a NVT ensemble. The constraint algorithm used was LINCS, which was applied to all bonds and angles in the protein.[19] The cut-off for non-bonded short-range interaction was set to 1.0 nm with the Verlet cut-off scheme. Long-range electrostatics were set to PME. The temperature coupling was set between the protein and the non-protein entities by using a Berendsen thermostat, with a time constant of 0.1 ps and the temperature set to reach 300 K with the pressure coupling off. Pressure equilibration was run at 300 K with a Parrinello-Rahman barostat on and set to 1 bar[20] in a NPT ensemble. The equilibration trajectories were set to 1 ns (discarded from the analysis), and the production MD simulations were performed for 100 ns.

Analysis of the trajectories was performed using GROMACS tools, focusing on the cluster analysis and the analysis of dihedrals.

1.3 Energy calculations

Single-point DFT calculations of compounds **A-C** were performed in Gaussian09[21] and Gaussview[22] using the 6-31G basis set, *in vacuo*. In parallel, potential energies were calculated using MM energy minimization using the setup described in 1.2, except the maximum force was increased from 1000 kJ/mol/nm to 10000 kJ/mol/nm, since both **16**-EGFR and **17**-EGFR are derived from crystal structures, do not want their structures to undergo significant changes during the energy minimization process.

1.4 Well-tempered metadynamics

After the structural alignment, it was found that the crystal structures of **16**-EGFR and **17**-EGFR overlapped well except for the pyrrolidine and the acrylamide moieties. This suggests that the dihedral angles involving the ether group connecting quinazoline to pyrrolidine could be responsible for the differences in IC_{50}. Therefore, to explore

the thermodynamic preferences for variations in the phi (quinazoline side) and psi (pyrrolidine side) angles of the ether group, we used Plumed 2.90[23] to conduct well-tempered metadynamics simulations on WT-EGFR and WWT-EGFR. In the simulations, the phi and psi of the ether group were used as collective variables, and three 100 ns simulations were run for each. Within the metadynamics simulations, the initial height of each Gaussian potential (HEIGHT) was set to 1.2 kJ/mol, with a width (SIGMA) of 0.05 nm, and a Gaussian was added every 1000 simulation steps (PACE). A bias factor (BIASFACTOR) of 10 was established to realize a well-tempered sampling strategy. The addition of Gaussians was based on the current value of the collective variable and was recorded in the HILLS file, to facilitate subsequent analysis and reproduction of the simulation process. Free energy profiles were obtained by integrating data collected during the metadynamics simulations. These free energy profiles displayed the variation in free energy at different angles of the ether group's phi and psi. By comparing the free energy associated with the dihedral angles of the **16**-EGFR crystal structure to those of adduct-prone conformations, we assessed the stability of **16**-EGFR under different dihedral angle states.

Crystallographic Table

Supplementary Table 1. X-ray data collection and refinement statistics

Commented [MM1]: Re-refined the two structures. Re-deposited them with the pdb

	WT EGFR complex 16 (pdb 9FZS)	WT EGFR complex 17 (pdb 9FZR)
**Data collection**		
Space group	I23	I23
Unit cell (Å)	a=b=c=145.097	a=b=c=145.823
Resolution (Å) (highest resolution shell)	59.24-2.12 (2.18-2.12)	59.53-1.99 (2.04-1.99)
Total observations	1173688 (99494)	1453649 (105527)
Unique	28920 (2385)	35412 (2491)
R_{merge}	0.103(4.960)	0.101(5.435)
Mean I/σ(I)	23.2 (1.0)	24.0 (0.9)
Multiplicity	40.6 (41.7)	41.0 (42.4)
Completeness %	100 (100)	100 (100)
CC(1/2)	1.00 (0.437)	1.00 (0.396)
**Refinement**		
Number of atoms (B-factor)<br>Protein<br>Other<br>Water	 5167 (76.9)<br>51 (77.5)<br>161 (70.5)	 5266 (66.9)<br>61.7 (50)<br>215 (69.7)
R (highest resolution shell)	0.194	0.186
R_{free} (highest resolution shell)	0.237	0.233
Rmsd bonds (Å)	0.0067	0.0082
Rmsd angles (°)	1.597	1.723

The structures have been deposited in the PDB with accession codes 9FZR & 9FZS.

References

1. P. G. Baraldi, R. Romagnoli, M. del Carmen Nuñez, M. Perretti, M. J. Paul-Clark, M. Ferrario, M. Govoni, F. Benedini, E. Ongini, *Journal of Medicinal Chemistry* 2004, 47, 711-719.
2. Kerong et al. Novel artemisinin derivatives and preparation method and use thereof CN103570738 (A) — 2014-02-12.
3. T.-B. Yu, J. Z. Bai, Z. Guan, *Angewandte Chemie International Edition* 2009, 48, 1097-1101.
4. K. D. Carey, A. J. Garton, M. S. Romero, J. Kahler, S. Thomson, S. Ross, F. Park, J. D. Haley, N. Gibson, M. X. Sliwkowski, *Cancer Research* 2006, 66, 8163-8171.
5. V. A. Saura, I. M. Burguete, F. Galindo, S. V. Luis, *Organic & Biomolecular Chemistry* 2017, 15, 3013-3024.
6. B. Barlaam, J. Anderton, P. Ballard, R. H. Bradbury, L. F. A. Hennequin, D. M. Hickinson, J. G. Kettle, G. Kirk, T. Klinowska, C. Lambert-van der Brempt, C. Trigwell, J. Vincent, D. Ogilvie, *ACS Med. Chem. Lett.* 2013, 4, 8, 742–746

7. *a)* K. C. Bang, Y. H. Moon, Y. K. Chang, Method for preparing 1-(4-(4-(3,4- dichloro-2-fluorophenylamino)-7-methoxyquinazolin-6-yloxy) piperidin-1-yl)-prop-2-en-1-one hydrochloride and intermediates used therein. US201214349887 20121005, October 5, 2014; b) M. Brindisi, G. Borrelli, S. Brogi, A. Grillo, S. Maramai, M. Paolino, M. Benedusi, A. Pecorelli, G. Valacchi, L. Di Cesare Mannelli, C. Ghelardini, M. Allarà, A. Ligresti, P. Minetti, G. Campiani, V. di Marzo, S. Butini, S. Gemma, *ChemMedChem* 2018, 13 (19), 2090–2103.
8. https://www.biosolveit.de/SeeSAR/
9. N. Schneider, G. Lange, S. Hindle, R. Klein, M. Rarey, *J Comput Aided Mol Des.* 2013, 27 (1), 15-29.
10. E.F. Pettersen, T.D. Goddard, C.C. Huang, G.S. Couch, D.M. Greenblatt, E.C. Meng, T.E. Ferrin. *J Comput Chem.* 2004, 25 (13), 1605-12.
11. B. Webb, A. Sali, *Methods Mol Biol.* 2017, 1654, 39-54
12. C. C. Huang, E. C. Meng, J. H. Morris, E. F. Pettersen, T. E. Ferrin, *Nucleic Acids Res.* 2014, 42 (Web Server issue): W478-84.
13. A. W. Sousa da Silva, W. F. Vranken, *BMC Res Notes.* 2012, 23, 5-367.
14. J. Wang, R. M. Wolf, J. W. Caldwell, P. A. Kollman, D. A. Case, *J Comput Chem*, 2004, 25, 1157-1174.
15. A. Jakalian, D. B. Jack, C. I. Bayly, *J Comput Chem.* 2002, 23 (16), 1623-41.
16. D. van der Spoel, E. Lindahl, *et al,* and H. J. Berendsen*, J. Comput. Chem.* 2005, 26, 1701–1718.
17. K. Lindorff-Larsen, S. Piana, K. Palmo, P. Maragakis, I. L. Klepeis, R. O. Dror, D. E. Shaw, *Proteins.* 2010, 78 (8), 1950-8.
18. T. Darden, D. York, L. Pedersen, *J. Chem. Phys.* 1993, 98, 10089–10092.
19. B. Hess, H. Bekker, J. G. E. M. Fraaije, *J. Comput. Chem.* 1997, 18, 1463–1472.
20. M. Parrinello, A. Rahman, *J. Appl. Phys.* 1981, 52, 7182.
21. J. M. Frisch, GAUSSIAN09. Available online: https://gaussian.com/ (accessed on 22 March 2024).
22. R. Dennington, T. Keith, J. Millam, Gauss View, Version 5; Semichem Inc.: Shawnee, KS, USA, 2009.
23. The PLUMED consortium, *Nat Methods* 2019, 16, 670–673.
